# Norovirus Regulation of the Innate Immune Response and Apoptosis Occurs via the Product of the Alternative Open Reading Frame 4

**DOI:** 10.1371/journal.ppat.1002413

**Published:** 2011-12-08

**Authors:** Nora McFadden, Dalan Bailey, Guia Carrara, Alicia Benson, Yasmin Chaudhry, Amita Shortland, Jonathan Heeney, Felix Yarovinsky, Peter Simmonds, Andrew Macdonald, Ian Goodfellow

**Affiliations:** 1 Section of Virology, Department of Medicine, Imperial College London, London, United Kingdom; 2 University of Texas Southwestern Medical Center at Dallas, Dallas, Texas, United States of America; 3 Laboratory of Viral Zoonotics, Department of Veterinary Medicine, University of Cambridge, Cambridge, United Kingdom; 4 Centre for Immunology, Infection and Evolution, University of Edinburgh, Edinburgh, United Kingdom; 5 Institute of Molecular and Cellular Biology, Faculty of Biological Sciences, University of Leeds, Leeds, United Kingdom; Mount Sinai School of Medicine, United States of America

## Abstract

Small RNA viruses have evolved many mechanisms to increase the capacity of their short genomes. Here we describe the identification and characterization of a novel open reading frame (ORF4) encoded by the murine norovirus (MNV) subgenomic RNA, in an alternative reading frame overlapping the VP1 coding region. ORF4 is translated during virus infection and the resultant protein localizes predominantly to the mitochondria. Using reverse genetics we demonstrated that expression of ORF4 is not required for virus replication in tissue culture but its loss results in a fitness cost since viruses lacking the ability to express ORF4 restore expression upon repeated passage in tissue culture. Functional analysis indicated that the protein produced from ORF4 antagonizes the innate immune response to infection by delaying the upregulation of a number of cellular genes activated by the innate pathway, including IFN-Beta. Apoptosis in the RAW264.7 macrophage cell line was also increased during virus infection in the absence of ORF4 expression. *In vivo* analysis of the WT and mutant virus lacking the ability to express ORF4 demonstrated an important role for ORF4 expression in infection and virulence. STAT1-/- mice infected with a virus lacking the ability to express ORF4 showed a delay in the onset of clinical signs when compared to mice infected with WT virus. Quantitative PCR and histopathological analysis of samples from these infected mice demonstrated that infection with a virus not expressing ORF4 results in a delayed infection in this system. In light of these findings we propose the name virulence factor 1, VF1 for this protein. The identification of VF1 represents the first characterization of an alternative open reading frame protein for the calicivirus family. The immune regulatory function of the MNV VF1 protein provide important perspectives for future research into norovirus biology and pathogenesis.

## Author Summary

This report describes the identification and characterization of a novel protein of unknown function encoded by a mouse virus genetically similar to human noroviruses. This gene is unique to the mouse virus and occupies the same part of the genome that codes for the major capsid protein. The protein that we have described as virulence factor 1 (VF1) is found in all murine norovirus isolates, absent in all human strains but is indeed expressed during infection. Its expression enables MNV-1 to establish efficient infection of its natural host through interference with interferon-mediated response pathways and apoptosis. Our data would indicate that the VF1 protein is multi-functional with an ability to modulate the host's response to infection. Murine noroviruses are frequently used firstly as a model to study human norovirus replication and pathogenesis, studies hampered by their inability to replicate in cell culture. Secondly, persistent infection of laboratory animals with murine norovirus may affect other models of disease using experimental mice. The role of VF1 in infection and pathology in the differential outcome of infection is the source of continued research in our laboratory.

## Introduction

Collectively, the innate and adaptive immune systems result in a strong evolutionary pressure on pathogens to develop countermeasures to allow their continued existence. Therefore pathogens, including viruses, have evolved a multitude of mechanisms for evading the host response to infection, often by the expression of proteins that interfere with cellular antimicrobial response mechanisms [1]. The size of RNA virus genomes is thought to be limited by the error prone nature of the viral polymerase. As a likely direct consequence, RNA viruses have evolved a variety of mechanisms to increase the coding capacity of their genomes [2]. These include the use of ribosomal frameshifting where a proportion of translating ribosomes change the reading frame to produce proteins with common N-terminal but a different C-terminal from the read-through sequence [3]. Many viruses have also evolved to use a mechanism that creates overlapping reading frames through the use of two or more transcription initiation sites or translation start codons within the same RNA sequence [4], [5].

Murine norovirus (MNV) was identified in 2003 as a virus that caused a lethal infection in immunocompromised mice [6]. However, MNV is now known to be a widespread infectious agent of laboratory mice with a reported seroprevalence of 20-64% [7], [8]. MNV is currently the only norovirus which replicates efficiently in tissue culture, where it has a tropism for dendritic and macrophage cells [9]. The availability of immortalized macrophage cell lines such as the murine macrophage RAW264.7, has allowed significant advances to be made in understanding the life cycle of this virus. For the first time critical processes in the norovirus life cycle have been dissected e.g. the mechanism of tissue culture mediated attenuation of MNV-1 [10] , the requirement for dynamin II and cholesterol during virus entry [11], [12], the identification and functional requirement for RNA secondary structures in virus replication [13] and pathogenesis [14] as well as the induction of apoptosis during infection [15], [16]. In addition, MNV has allowed an unprecedented analysis of the immune response to norovirus infection [17],[18]–[21]. This broadening in understanding of norovirus replication has been facilitated greatly by the development of murine norovirus reverse genetics [22], [23] and its recent optimisation [24]. The role of murine norovirus in potential exacerbation or complication of other diseases, especially murine models of infection, has also been investigated. This is certainly warranted given the seroprevalence of MNV in animal houses. Studies with models of Crohn's disease [25] or bacterial induced inflammatory bowel disease [26] showed a significant impact of MNV and MNV infection prolongs the shedding of mouse parvovirus [27]. In contrast, MNV co-infection had little or no impact on murine CMV [28], Friend retrovirus infection [29] or models of diet induced obesity and insulin resistance [30]. These contrasts warrant further studies into the nature and mechanisms of interference observed in mouse models of disease. MNV has also provided a useful experimental system in determining the immune responses required for efficient norovirus vaccination [18],[31]. Collectively, this highlights both the relevance of MNV as a significant infectious agent in its own right and also the utility of MNV as a model for human norovirus. Continued research into what differentiates murine and human noroviruses and how norovirus infection affects the host cell is therefore of upmost importance to both fields of research.

**Figure 1:**
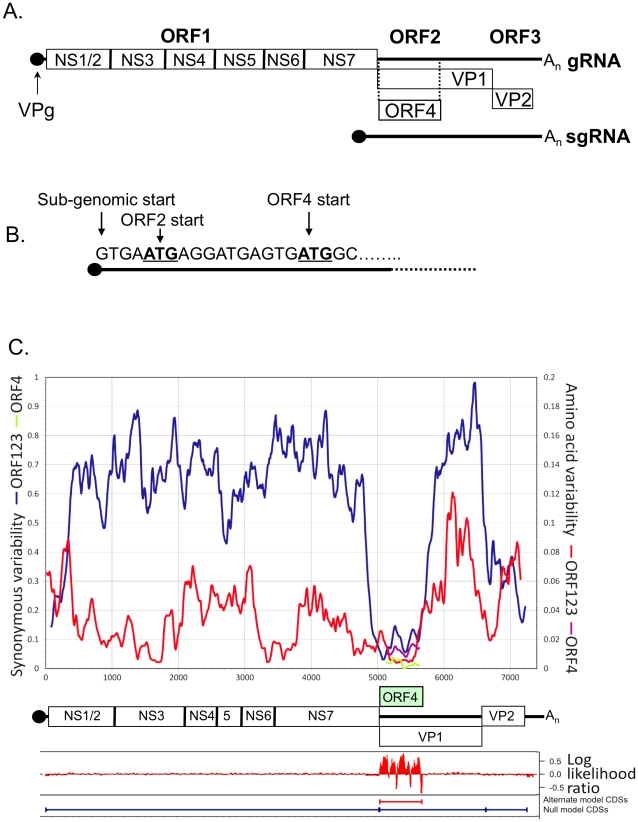
Schematic representation of the murine norovirus genome highlighting the position of ORF4. (A) The three previously described open reading frames (ORFs) are highlighted as ORF1, ORF2 and ORF3. The NS1-7 nomenclature of the mature peptides generated from ORF1 [as described by 86] is also shown. The position of the ORF4 reading frame is highlighted along with (B) the sequence of the 5′ end of the MNV sgRNA showing the start codons for VP1 and ORF4. (C) Synonymous and amino acid sequence variability scan of conventional MNV genes (ORF1, ORF2 and ORF3) with positions depicted to scale in genome diagram below axis. Position and variability of putative ORF4 superimposed on diagram. Log likelihood of alternate (ORF4) and null (ORFs 1, 2 and 3 only) coding predictions generated by MLOGD (mean values of 21 adjacent bases shown).

Unlike other members of the *Caliciviridae,* which typically encode three open reading frames [6], our analysis and that presented during large scale sequencing of many MNV genomes [32] indicates the presence of a fourth potential ORF in the MNV genome (Figure 1A) In this study we demonstrate that the protein encoded by ORF4 is expressed during virus infection, is not essential for virus replication in tissue culture but plays a role in viral virulence and therefore represents a novel viral virulence factor. Based on the findings that it possesses anti-innate immune activity, contribute towards the regulation of virus induced apoptosis during infection and modulates the outcome of experimental infection of mice, we have described the ORF4 gene product as virulence factor 1 (VF1). The study provides important insights into the mechanisms of norovirus avoidance of the innate immune response and norovirus pathobiology.

## Results

### Bioinformatic prediction and analysis of ORF4/VF1

The region of the genome encoding VF1 contains an intact reading frame in all available MNV sequences derived from different isolates or strains (Figure 1A, 1B and data not shown). In contrast to the typical 8–15% sequence divergence seen between MNV variants in the amino acid sequences of ORF1, ORF3 and the predicted single coding region of ORF2, variability is markedly suppressed in the predicted ORF4 and double coding region of ORF2 (3%; Table 1; Figure 1C). Almost all sequence variability between MNV variants in the single coding regions (ORF1 and ORF3) occurs at synonymous sites. dN/dS ratios, namely the substitution rates at non-synonymous and synonymous sites, ranging between 0.03–0.10 are indicative of strong negative selection. In the double coding region of ORF2 (i.e. the region which codes for both VP1 and VF1), the restricted variability that is observed occurs at synonymous sites in the ORF2 reading frame (dN/dS: 0.044) consistent with stronger sequence constraints in the conventional reading frame encoding the MNV structural protein than in the ORF4 gene (dN/dS ≈ 2). However, the elevated ratio relative to that of ORF2 arose through greater suppression of synonymous variability in this reading frame, rather than increased amino acid sequence variability. dN values were 0.04 and 0.03 in ORF2 and ORF4 respectively.

**Table 1:** Sequence divergence of MNV genes.

Mean pairwise distances^2^						
**Region**	**Coding**	**Position^1^**	**P**	**dS**	**dN**	**dN/dS**
ORF1	Single	6-5066	0.11	0.55	0.02	0.029
ORF2	Single	5710–6678	0.15	.063	0.04	0.064
ORF3	Single	6681–7304	0.08	0.28	0.03	0.103
ORF2	Double	5056–5706	0.03	0.09	0.00	0.044
ORF4/VF1	Double	5069–5707	0.03	0.02	0.03	1.959

1 Genome positions numbered as in MNV3 (DQ223042).

2 Juker-Cantor correction for multiple substitution applied to p (all sites), synonymous and non-synonymous distances.

As well as suppressing variability, the existence of a second reading frame in ORF2 leads to altered codon usage by the ORF4/VF1 coding sequence. For example, there was a significant overrepresentation of the UUG triplet coding for Leu in ORF4 (15 from 33, compared to 17 from 128 in ORF1; p<0.001 in a 6×2 contingency table for the 6 synonymous Leu codons), whereas there were no differences in Leu codon usage between ORFs 1, 2 (single coding region) and ORF3. The program MLOGD identifies overlapping coding sequences by specific codon usage signatures arising from mutational constraints consequent to the requirement to maintain protein function in two putative genes [33]. The relative likelihood that a given sequence region is single-coding or double-coding was calculated using a codon usage table and nucleotide mutation and amino acid substitution matrices (Figure 1C). This analysis provides independent support for the existence of ORF4/VF1, independent of its effect on sequence variability and evolutionary conservation. The first methionine codon in ORF4 at 5069 lies two residues downstream from a stop codon in that reading frame, and is 2 and 4 residues away from Met codons in ORF1 (including the -1 frameshift). The ORF4 start codon is in a strong Kozak context (G at +4 and -3) and likely represents the translation start site of VF1.

### *In vitro* translation of the MNV-1 subgenomic RNA produces three proteins, VP1, VP2 and VF1

**Figure 2:**
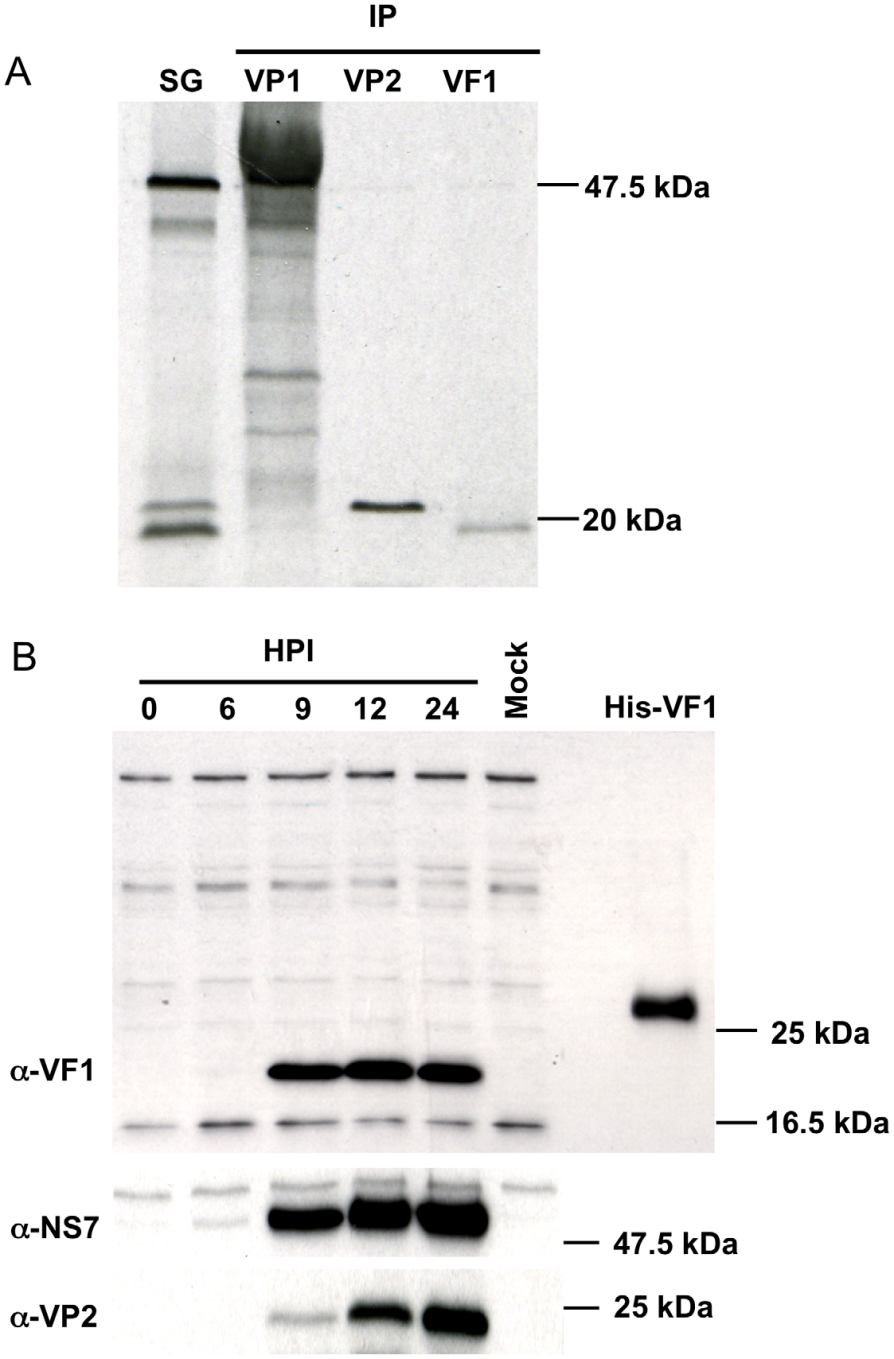
*In vitro* synthesis of the murine norovirus VF1 protein. (A) Coupled *in vitro* transcription and translation of a cDNA construct containing the murine norovirus 1 (MNV) sgRNA was performed prior to immunoprecipitation (IP) with polyclonal antisera to VP1, VP2 or VF1. Immunoprecipitated complexes were subsequently resolved by SDS-PAGE (15% polyacrylamide) alongside an aliquot of the complete reaction prior to immunoprecipitation (SG). (B) Western blot analysis indicating VF1 expression during virus high multiplicity infection. RAW264.7 cells were infected with MNV-1 at a MOI of 5 TCID_50_ per cell. Protein samples were prepared at various times post infections (HPI) and then separated by SDS-PAGE (15% polyacrylamide) prior to immune-blotting with antisera to VF1, NS7 or VP2.

The ability of the MNV-1 subgenomic RNA (sgRNA) to produce a protein from the open reading frame predicted to encode VF1 was examined by *in vitro* translation of a plasmid containing the entire MNV-1 sgRNA under control of a T7 RNA polymerase promoter (Figure 2A). A coupled transcription and translation reaction of the MNV-1 sgRNA produced three proteins and the identity of the major (VP1) and minor (VP2) capsid proteins were confirmed using immunoprecipitation (Figure 2A). Polyclonal antisera to a peptide from MNV-1 VF1 was generated in rabbits and used to confirm the identity of the VF1 protein product by immunoprecipitation (Figure 2A). Full length his-tagged VF1, purified from *E.coli* was poorly immunogenic, hence a modified immunization protocol that used a variety of forms of VF1 (described in Materials and Methods), followed by affinity purification was required in order to obtain reactive antisera. Immune sera from MNV-1 infected mice did not contain antibodies to VF1 as determined by western blot using recombinant his tagged VF1 (data not shown).

### VF1 is produced during MNV-1 replication in tissue culture

To examine the expression of VF1 during MNV-1 replication in tissue culture, the well established RAW264.7 cell culture system for MNV [9] was used and the production of VF1 analyzed by western blot (Figure 2B). Using a high multiplicity of infection (MOI of 5 TCID_50_/cell) infection, VF1 was readily detected as early as 9 hours post infection, appearing at the same time as the minor capsid protein VP2 (Figure 2B). In contrast, the viral RNA polymerase NS7 was detected as early as 6 hours post infection (Figure 2B). Whilst we were unable to detect VF1 and VP2 prior to 9 hours, this may simply be a reflection of the sensitivity of the antisera used in the assay, but may also reflect the kinetics of viral sgRNA synthesis, as this is likely to occur after the initial rounds of viral genomic RNA synthesis. VF1 and VP2 expression levels observed over the course of the infection were also significantly different, with VF1 being expressed to a higher degree than VP2. Whilst this may be a reflection of the differences in the ability of the antisera to detect both proteins, it is known that VP2 synthesis requires translation re-initiation at the end of VP1 [34] which is likely to produce reduced levels of VP2 relative to the other proteins expressed from the viral sgRNA.

### VF1 is not essential for MNV-1 replication in tissue culture

**Figure 3:**
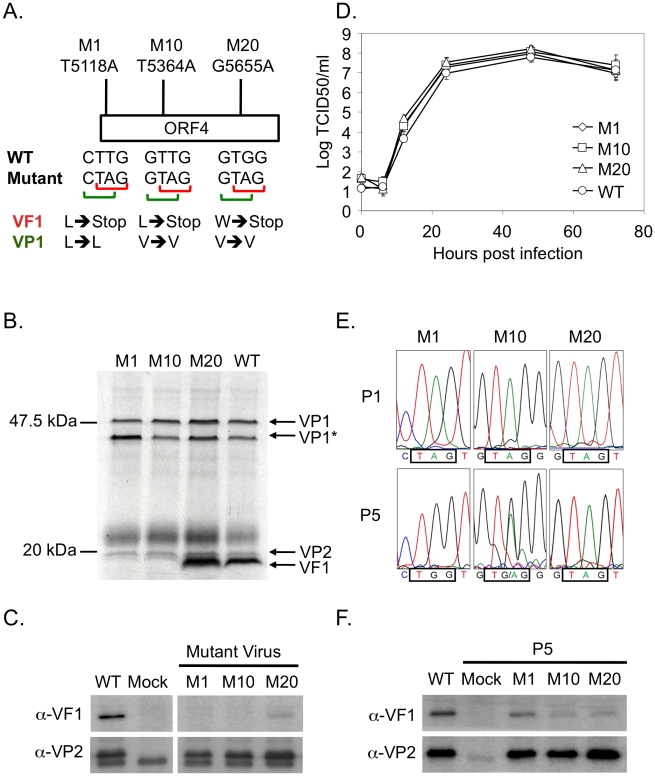
VF1 is not required for murine norovirus replication in tissue culture although there is a ‘fitness cost’ to the virus. (A) Schematic representation of the position and nucleotide changes introduced to generate the VF1 mutant viruses M1, M10 and M20. Nucleotide numbers refer to the positions in the MNV-1 genome. The effect of the introduced nucleotide changes on the VF1 and VP1 protein sequences are also illustrated. Note that the mutations introduced in M1, M10 and M20 do not affect the coding sequence of the capsid protein VP1 but introduce a stop codon into VF1 at various positions. (B) Coupled *in vitro* transcription and translation reactions confirming the lack of VF1 expression in M1 and M10 viruses, with M20 producing a marginally shorter VF1 product. PCR products containing the subgenomic RNA region under control of a T7 RNA polymerase promoter were generated from cDNA constructs of either wild-type (WT), M1, M10 or M20 VF1 mutants and subsequently used for transcription and translation (TNT) *in vitro* in the presence of S35 methionine. Radioactively labeled proteins were subsequent resolved by SDS-PAGE, prior to exposure to film. VP1* represents a potential shorter VP1 product generated by translation initiation from an AUG initiation codon in-frame yet downstream from the authentic VP1 AUG. (C) Western blot analysis of RAW264.7 cells infected with low passage, sequence verified stocks of either wild-type (WT), M1, M10 or M20 VF1 mutant viruses. RAW264.7 cells were infected at a MOI 10 TCID_50_ per cell and harvested 12 hours post infection, prior to separation by SDS-PAGE and western blot using either anti-VF1 or anti-VP2 antisera. (D) Multi-cycle growth kinetics analysis of VF1 mutant viruses M1, M10 and M20 in RAW264.7 cells. Cells were infected with a MOI of 0.01 TCID_50_ per cell and samples harvested at various times post infection prior to titration on RAW264.7 cells. Virus yield is expressed as TCID_50_/ml. Infections were performed in triplicate, with the average virus titer and standard deviation plotted. (E) Sequence chromatograms of M1, M10 and M20 VF1 mutant viruses after passage 1 or 5 in RAW264.7 cells. Viruses obtained from passage 1 and 5 low multiplicity infections (MOI) of RAW264.7 cells were used to infect a subsequent monolayer at high MOI prior to RNA isolation, RT-PCR amplification of the region encompassing ORF4 and sequence analysis. The positions of the introduced stop codons in the mutants M1, M10 and M20 are boxed as are the sequences after 5 repeated passages in cell culture. (F) Western blot analysis of VF1 and VP2 expression in cells infected with either wild-type MNV or passage 5 VF1 mutant viruses M1, M10 and M20. 18 hours post infection at a high MOI (4 TCID_50_ per cell) cells were harvested, separated by SDS-PAGE prior to western blot analysis using antisera to either VF1 or VP2. Note that batch-to-batch variation in the quality of the anti-VP2 antisera accounts for the variations in the levels of non-specific proteins detected in panels C and F.

To determine if VF1 was required for MNV-1 replication in tissue culture we used a recently developed reverse genetics system [22] to truncate the VF1 coding region at various positions. Three mutants were created containing single nucleotide changes that lead to the introduction of a stop codon in the VF1 coding region but which did not alter the VP1 coding sequence (Figure 3A): M1 containing the mutation T5118A truncating the VF1 protein at amino acid 16; M10 containing the mutation T5364A, truncating VF1 at amino acid 98; M20 containing the mutation G5655A, truncating VF1 at amino acid 195. All mutations were introduced at positions where it was possible to change the VF1 coding sequence without affecting the major capsid protein VP1. Single nucleotide substitutions were used due to the nature of the overlapping coding regions. The interruption of VF1/ORF4 was confirmed by *in vitro* coupled transcription and translation of a PCR product encompassing the sgRNA of each mutant compared to wild-type MNV-1 (Figure 3B). VF1 was readily detected after *in vitro* translation of the wild type sgRNA product as well as the sgRNA from the M20 mutant that encodes a C-terminally truncated form of VF1. VF1 was not detected after *in vitro* translation of the sgRNA from either the M1 or M10 VF1 truncations as expected (Figure 3B). Recovery of wild-type and VF1 mutant viruses was performed using fowlpox mediated expression of T7 RNA polymerase to drive the synthesis of MNV-1 RNA in cells transfected with full length cDNA constructs of MNV-1 as described [22]. As we have previously reported, the BHK cell line used during virus recovery, although permissive to virus replication, cannot be infected with MNV due to the lack of a suitable receptor [22], therefore the yield of virus from this system represents a single round of virus replication only. The initial yields of VF1 knockout or truncation viruses were comparable to that derived from wild-type cDNA (∼1–5×10^4^ TCID_50_ per 35mm dish, data not shown), indicative that VF1 was not required for virus replication in tissue culture. Western blot analysis of cells infected with the sequence verified M1, M10, M20 viruses confirmed that VF1 was not expressed in cells infected with either M1 or M10, but low levels of VF1 were observed in M20 infected cells (Figure 3C). The levels of VP2 produced by the VF1 knockout viruses were comparable to the wild-type MNV-1 derived from cDNA, confirming comparative levels of infection (Figure 3C). It is possible that the truncation of VF1 in the mutant M20 results in some protein misfolding, decreasing the half-life of the resulting truncated protein. The growth kinetics of low passage, sequence verified M1, M10 and M20 viruses was examined by both single-step (data not shown) and multi-step growth curve analysis and were indistinguishable from that of the wild-type parental MNV-1 derived from cDNA (Figure 3D), indicating that VF1 is not required for MNV-1 replication in tissue culture.

### Lack of VF1 results in a ‘fitness cost’ in tissue culture

The observation that all MNV isolates identified to date retain ORF4/VF1 and that repeated passage of wild-type virus in tissue culture does not result in the loss of VF1 (data not shown), indicates that although VF1 is not essential for virus replication in tissue culture, it confers some benefit to virus replication. To address this, we examined the stability of the mutations in the M1, M10 and M20 viruses following repeated low multiplicity of infection (0.01 TCID_50_ per cell), multi-cycle replication in tissue culture. We observed that the mutations M1 and M10 were under negative selection in tissue culture whereas M20 was stable (Figure 3E). Sequence analysis of the virus population after 5 low multiplicity, multicycle passages in tissue culture, subsequent to the initial amplification after reverse genetics recovery, demonstrated that the M1 virus, which at passage 1 contained the mutation T5118A introducing a stop codon at position 17 in VF1, had introduced the mutation A5118G by the 5^th^ additional passage, restoring full-length VF1 production by the insertion of a tryptophan residue. Analysis of the M10 virus population, which had the mutation T5364A at the first passage, also indicated that the population was heterogeneous and that in a proportion the VF1 open reading frame was restored by the introduction of the mutation A5364G. As with the M1 virus, this mutation is predicted to result in the introduction of a tryptophan at position 99. In contrast however, sequence analysis of the M20 virus after repeated multicycle passage in tissue culture demonstrated that the introduced mutation (G5655A) was in fact stable (Figure 3E), which may indicate that the major functional domain lay within the 195 amino acids. Western blot analysis of cells infected with ‘passage 5’ stocks of M1 and M10 viruses indicated that, as expected from sequence analysis, VF1 expression was detectable (Figure 3F), although the levels were notably lower than observed in WT infected cells. This reduced level may be in part due to the effect of the amino acid change on VF1 protein stability, but clearly for the M10 virus population the heterogeneous nature of the M10 virus stock (Figure 3E) is likely a contributing factor. M20 virus stocks maintained the ability to express low levels VF1 as previously seen using the initial virus stocks (Figure 3C and 3F). In all cases, the level of virus replication was similar as determined by the expression of the minor capsid protein VP2 (Figure 3F) and virus titre (data not shown).

### VF1 localizes to mitochondria

**Figure 4:**
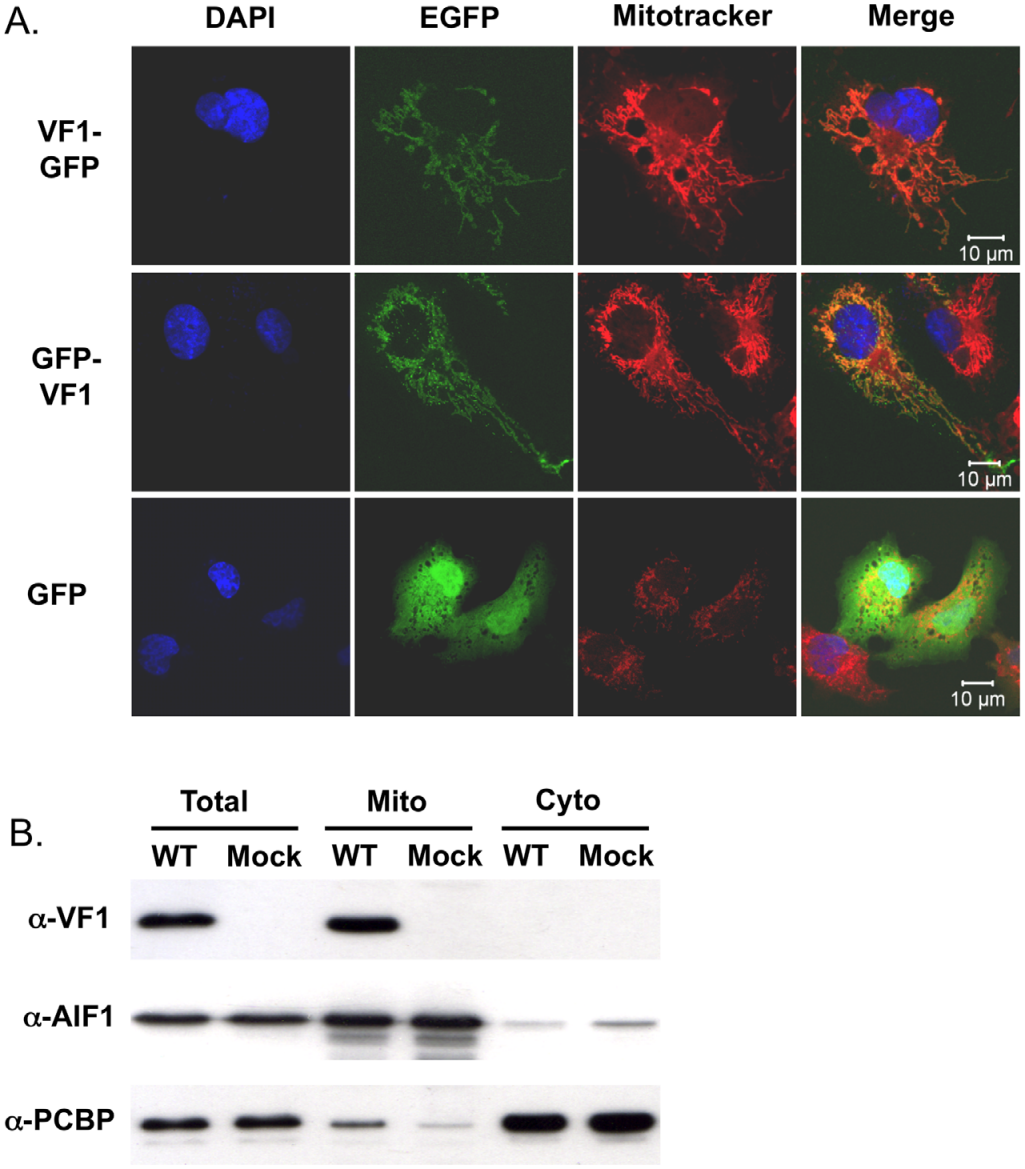
VF1 localizes to mitochondria. (A) Confocal microscopy of cells transfected with either N- or C-terminal fusions of EGFP to VF1. Cos7 cells were transfected with plasmids expressing VF1-EGFP fusion proteins, prior to co-staining with Mitotracker, fixation and staining with DAPI. Cells were analyzed using a Zeiss Meta510 confocal microscope. GFP transfected and Mitotracker stained Cos7 cells are also shown as a control. (B) Biochemical fractionation demonstrates that VF1 localizes to mitochondria during virus infection. RAW264.7 cells were either mock infected or infected with wild-type MNV followed by mitochondrial purification 12 hours post infection. Samples were subsequently analyzed by western blot using antisera to VF1, apoptosis inducing factor 1 (AIF) or poly rC-binding protein1/2 (PCBP). Total cellular lysate was prepared by lysing cells directly in SDS-PAGE sample buffer and run alongside as a control.

To gain further insights in the potential function of VF1, the localization of VF1 was examined by confocal microscopy. Due to the high degree of cross-reactivity of the VF1 antisera with endogenous host cell proteins (Figure 2B), fusions of MNV-1 VF1 to EGFP were used to examine VF1 localization in cells. Transfection of COS7 cells with cDNA constructs expressing either N or C-terminal fusions of MNV-1 VF1 with EGFP demonstrated a pattern of EGFP expression characteristic of mitochondrial localization (Figure 4A). This was confirmed via co-staining of cells with the mitochondrial vital stain Mitotracker (Invitrogen) (Figure 4A). Similar co-localization of VF1-GFP and mitochondria was observed in BHK and 293 cells (data not shown). The expression levels observed in cells transfected with the VF1-GFP fusion proteins were substantially lower than those observed in infected cells as expression was not detectable by western blot analysis with either α-VF1 or α-GFP antisera (data not shown).

To confirm the mitochondrial localization of VF1 during virus infection, mitochondria were purified from infected RAW264.7 cells at 15 hours post infection and analyzed for the presence of VF1 by western blot (Figure 4B). Whereas the well characterized host cell nucleic acid binding proteins PCBP1/2 were shown to be predominantly cytoplasmic as expected [35], VF1 was only detected in the mitochondrial fraction (Figure 4B). Apoptosis inducing factor 1, a predominantly mitochondrial protein was enriched in the mitochondrial fraction, confirming the validity of the purification procedure (Figure 4B).

### VF1 production affects mitochondrial-dependent innate immune signalling

**Figure 5:**
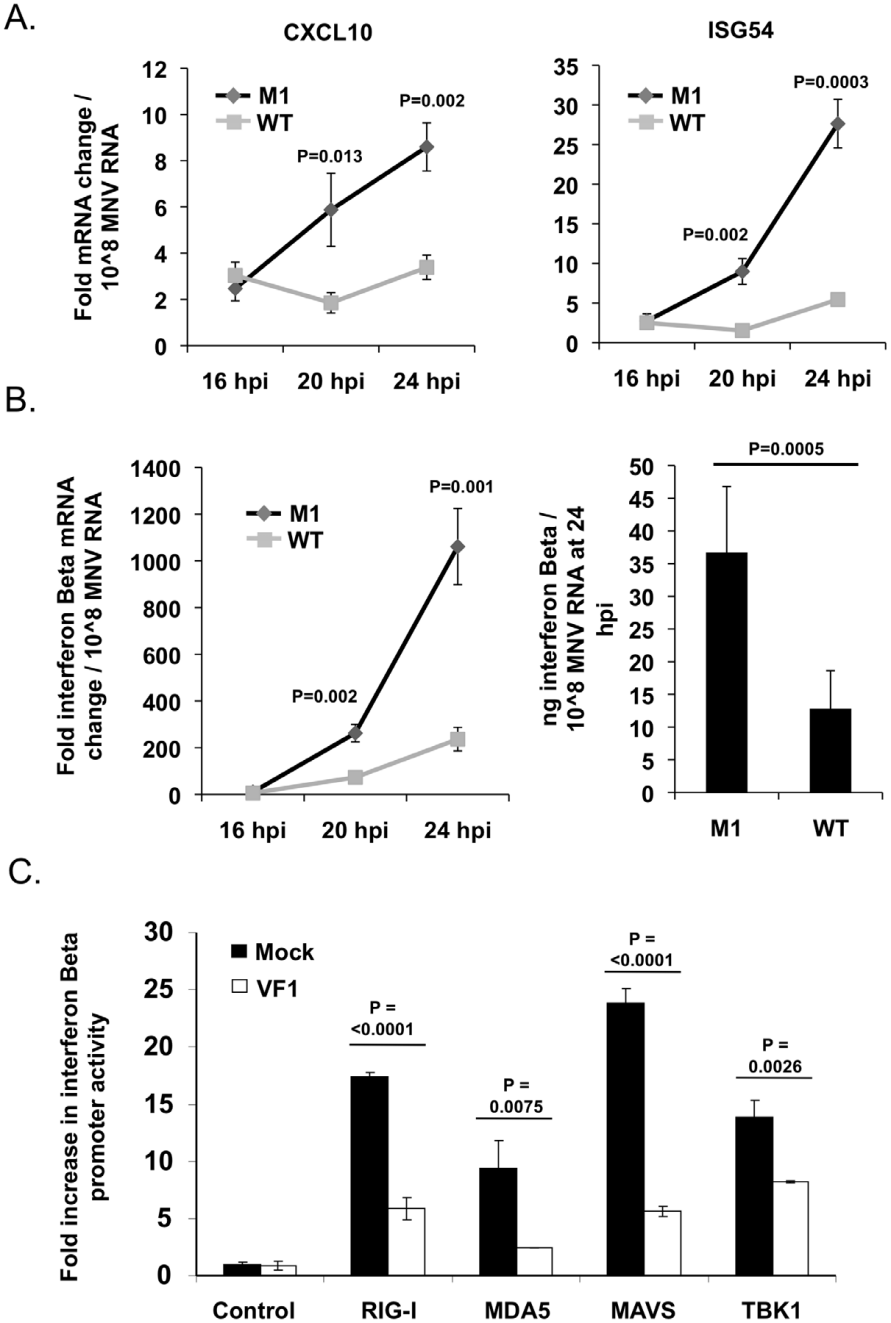
VF1 antagonizes the innate immune response to MNV infection. (A) Time course of ISG54 and CXCL10 mRNA expression in RAW264.7 cells infected at an MOI of 0.1 TCID_50_ per cell. mRNA levels were quantified by qPCR using an endogenous control gene (Hypoxanthine-guanine phosphoribosyltransferase, HPRT). Expression of the respective mRNAs was then calculated using the ΔΔCt method to compare infected and mock infected cells. Relative fold change was calculated using mock infected samples taken at comparable time points. Normalization was performed following MNV quantification in each sample. Infections were carried out in triplicate with each sample being subsequently analyzed in duplicate by qPCR. The error bars denote standard deviation from the mean. Statistical analysis was performed using an unpaired two tailed t test. (B) Time course of IFN-Beta mRNA and protein production in RAW264.7 cells infected as in (A). mRNA upregulation was calculated as described for (A). Interferon Beta protein was quantified by murine IFN-B specific ELISA at 24 hpi from supernatant samples taken from infected cells. For the ELISA infections were carried out in sextuplate. The error bars denote standard deviation from the mean. (C) MEF cells transfected with an IFN-Beta promoter driven luciferase reporter as well as expression constructs for RIG1, MDA5, MAVS or TBK1 were co-transfected with plasmid DNA expressing VF1 or blank message. Luciferase production was assayed 24 hours post transfection as described in the Materials and Methods. Transfections were carried out in triplicate. The error bars denote standard deviation from the mean. Statistical analysis was performed using an unpaired two tailed t test.

RNA viruses frequently encode proteins that antagonize the innate immune response to infection. Mitochondria play a significant role in signaling innate immune responses through the well characterized mitochondrial antiviral signaling protein (MAVS), an integral membrane protein found in the outer mitochondrial membrane [36]–[39]. MAVS is a key adapter protein in the sensing of viral RNA by RIG-I and MDA5 that, in part, leads to IRF3 and NFΚB activation and the upregulation of antiviral genes such as IFN-Beta, CXCL10 and ISG54 [37]. Given the mitochondrial localization of VF1 in infected cells we assessed the activation of this sub-section of the innate immune response in both M1 and WT infected cells. RAW264.7 cells infected at a low MOI (0.1 TCID_50_ per cell) with the M1 VF1 knockout virus exhibited a greater induction of antiviral genes such as ISG54, CXCL10 and IFN-Beta in response to viral infection than those infected with the WT virus (Figure 5A and Figure 5B). Alterations to the levels of mRNA were calculated relative to uninfected cells using the standard ΔΔCT method with hypoxanthine phosphoribosyltransferase 1 (HPRT) (Figure 5A and Figure 5B) or actin (data not shown) mRNA levels used as endogenous controls. CXCL10, ISG54 and IFN-Beta mRNA levels were then normalized to the amount of viral RNA present in each sample in order to calculate the rate of induction of the innate immune response over time. This method of data normalization was also used to overcome variations often observed in the rate of virus replication seen in a variety of RAW264.7 cell clones (not shown). Normalizing the mRNA fold change to a constant amount of MNV RNA established that in all cases examined (CXCL10, ISG54 and IFN-Beta) the M1 infection causes a much more rapid induction of the innate immune response. For instance CXCL10 in M1 infected cells is induced 15.5 fold more quickly in response to the same amount of viral RNA than in WT infected cells. This value is calculated by comparing the slope/gradient for M1 and WT (Figure 5A) which represents the rate of induction of each gene. Significantly, the IFN-Beta and ISG54 mRNAs are also activated more quickly, 4.6 and 8.3 fold respectively, in M1 infected cells compared to WT equivalents. Of note, the total fold increase in CXCL10, ISG54 and IFN-Beta mRNA induced in M1 infected cells was significantly higher than that observed in WT cells at both 20 and 24 hours post infection (Figure 5A). These time points, and the eight hour window from 16 to 24 hpi, reflect the period of amplification for innate immune related gene activation following low MOI MNV-1 infection of RAW264.7 cells, since quantification of mRNA fold change at 16 hpi showed little or no increase (Figure 5A and 5B). The activation of the IFN-Beta mRNA in infected cells was also shown to correlate to increased protein production and secretion using ELISA (Figure 5B). The amount of IFN-Beta protein in the supernatants of infected RAW264.7 cells was significantly higher in M1 than WT infected cells at 24 hours post infection (Figure 5B). Protein production was again normalized to a constant level of viral RNA to demonstrate the relative response to WT and M1 replication.

Treating cells with poly (I:C), and analysis of gene expression, confirmed the sensitivity of RAW264.7 cells to dsRNA over an equivalent time course. Induction of ISG54, CXCL10 and IFN-Beta was demonstrated in poly (I:C) treated cells confirming their suitability for the investigation of innate immune responses to RNA stimuli (Figure S1). In addition, UV inactivated M1 and WT virus showed no significant induction of ISG54, CXCL10 and IFN-Beta when used in equivalent experiments and compared to mock infected cells (Figure S1). The IFN-Beta protein secretion in response to poly (I:C) and UV inactivated viruses was equivalent to that seen for the mRNA (Figure S1).

This ability of VF1 to antagonize the innate immune response was confirmed independently of infection using an IFN-Beta promoter driven luciferase assay. Murine embryonic fibroblast (MEF) cells were co-transfected with plasmid DNA expressing firefly luciferase under the control of an IFN-Beta promoter as well as expression constructs for RIGI, MDA5, MAVS or TBK1 whose ectopic over-expression has been shown to drive IFN-Beta production [40]. In addition these cells were co-transfected with either the empty vector or a plasmid expressing the MNV-1 VF1 protein. Over-expression of RIG1, MDA5, MAVS and TBK1 in cells transfected with the IFN-Beta promoter driven reporter resulted in an expected increase in luciferase production in all cases. However, this induction was significantly reduced in all instances where VF1 was co-transfected in comparison to the empty vector (Figure 5C). This indicates that VF1 in some way antagonizes the induction of IFN-Beta, correlating with the results observed in infected RAW264.7 cells.

### VF1 plays a role in controlling virus-induced apoptosis

**Figure 6:**
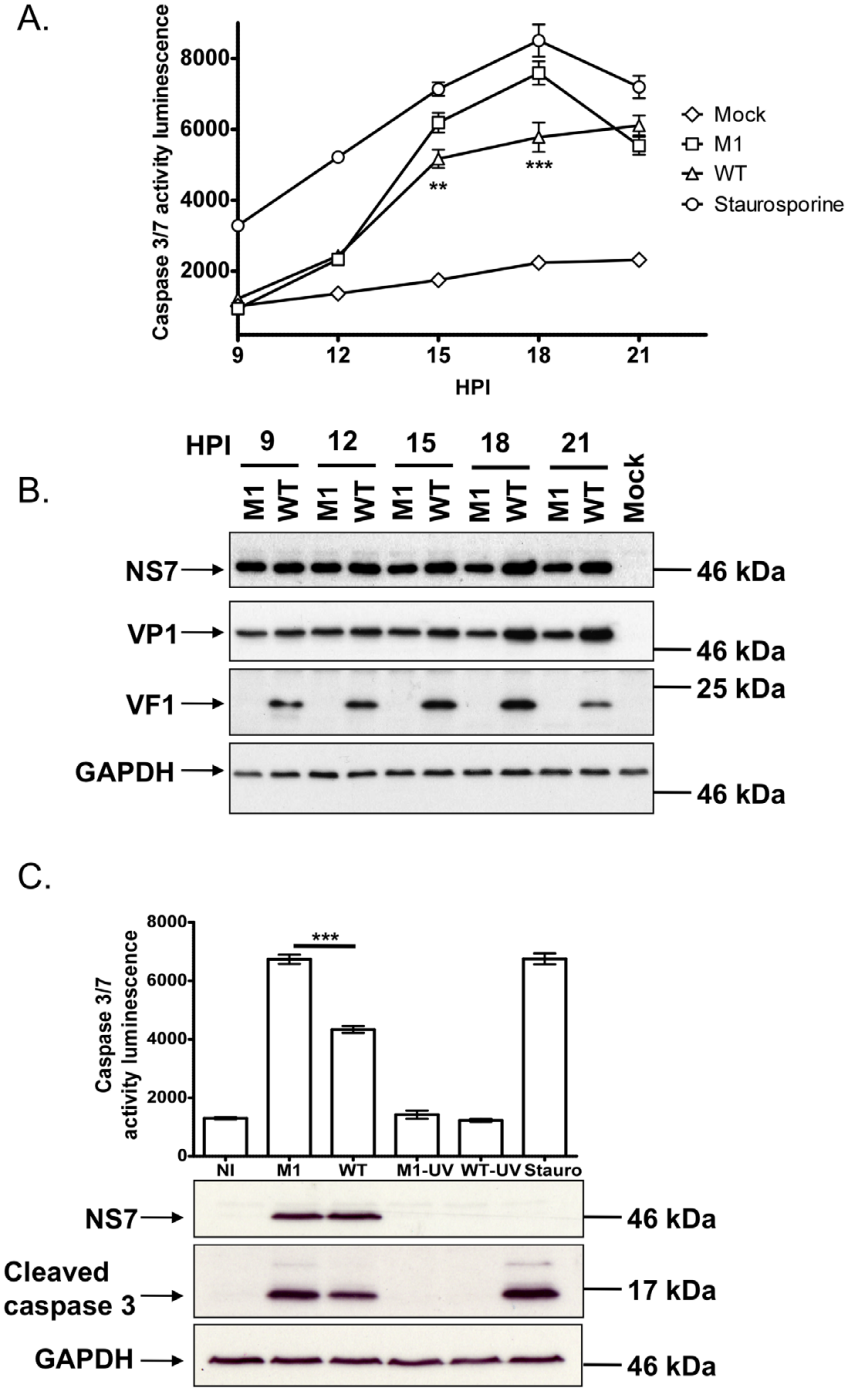
Viruses lacking VF1 are altered in their ability to induce apoptosis. (A) Time course of caspase 3/7 activity in RAW264.7 cells treated with Staurosporine or infected with wild-type MNV-1 (WT) or a VF1 knockout virus (M1). Cells were infected at a MOI of 5 TCID_50_ per cell and samples harvested at various times post infection. Caspase 3/7 activity was determined using a commercial assay from Promega (Glo 3/7) and the activity represented as relative light units (RLU). Each assay was performed in triplicate with the average and standard error within a single experiment plotted. Statistical analysis was performed using 2 way ANOVA with Bonfferoni post tests (B) Western blot analysis of extracts prepared from cells infected as detailed in (A). Samples were prepared at various times post infection, separated by SDS-PAGE and subsequently immune blotted using antisera to GAPDH, NS7, VP1 or VF1. Non-infected (Mock) cells were included as controls. (C). Caspase 3/7 activity assay in RAW 264.7 cells infected with M1 or WT MNV-1 or UV-inactivated M1 and WT viruses. Samples were analyzed at 16 hours post infection with triplicate samples taken. The mean and standard error within a single experiment are plotted. Statistical analysis was performed using an unpaired two tailed t-test (WT versus M1). P<0.01 is illustrated as ** and P<0.001 is shown as ***. Western blot analysis of GAPDH, NS7 and cleaved caspase 3 for the same samples is also shown.

MNV-1 infection is known to result in the induction of apoptosis via the down regulation of survivin, activation of caspases [41], as well as induction of cathepsin B activity [16]. Given our observation that VF1 localized with mitochondria and the key role mitochondria play in regulating apoptosis, we also examined how the absence of VF1 during infection affected the activity of the executioner caspases 3 and 7. Proteolytic activation of caspase 3 and 7, both of which play critical roles in the induction of apoptosis via the intrinsic cellular pathway, was also investigated (Figure 6A and 6C). As previously described [16], [41], a rapid increase in caspase 3/7 activity was observed in cells infected with wild type MNV-1 from 12 hours onwards (Figure 6A). Cells infected with MNV-1 lacking VF1 (M1), displayed significantly higher caspase 3/7 activity at 15 and 18 hours than cells infected with wild type MNV-1 (Figure 6A). Cells infected with the M1 virus lacking VF1 also displayed increased levels of the cleaved caspase 3 at 16 hours post infection (∼50% more than WT when quantified by densitometry) as determined by western blot analysis (Figure 6C). There was a notable alteration to the kinetics of viral protein production in the later stages of virus infection; whereas VP1 and NS7 levels continue to increase from 15 hours onwards in cells infected with WT MNV-1, the levels observed in cells infected with the M1 virus remained largely constant (Figure 6B). Importantly, the levels of infectious virus produced during infection were identical (Figure 3D). Induction of caspase 3/7 activities was shown to be due to virus replication as prior virus inactivation by UV treatment prevented virus induced caspase activity and the appearance of cleaved caspase 3 (Figure 6C).

### VF1 contributes to virus replication *in vivo*

**Figure 7:**
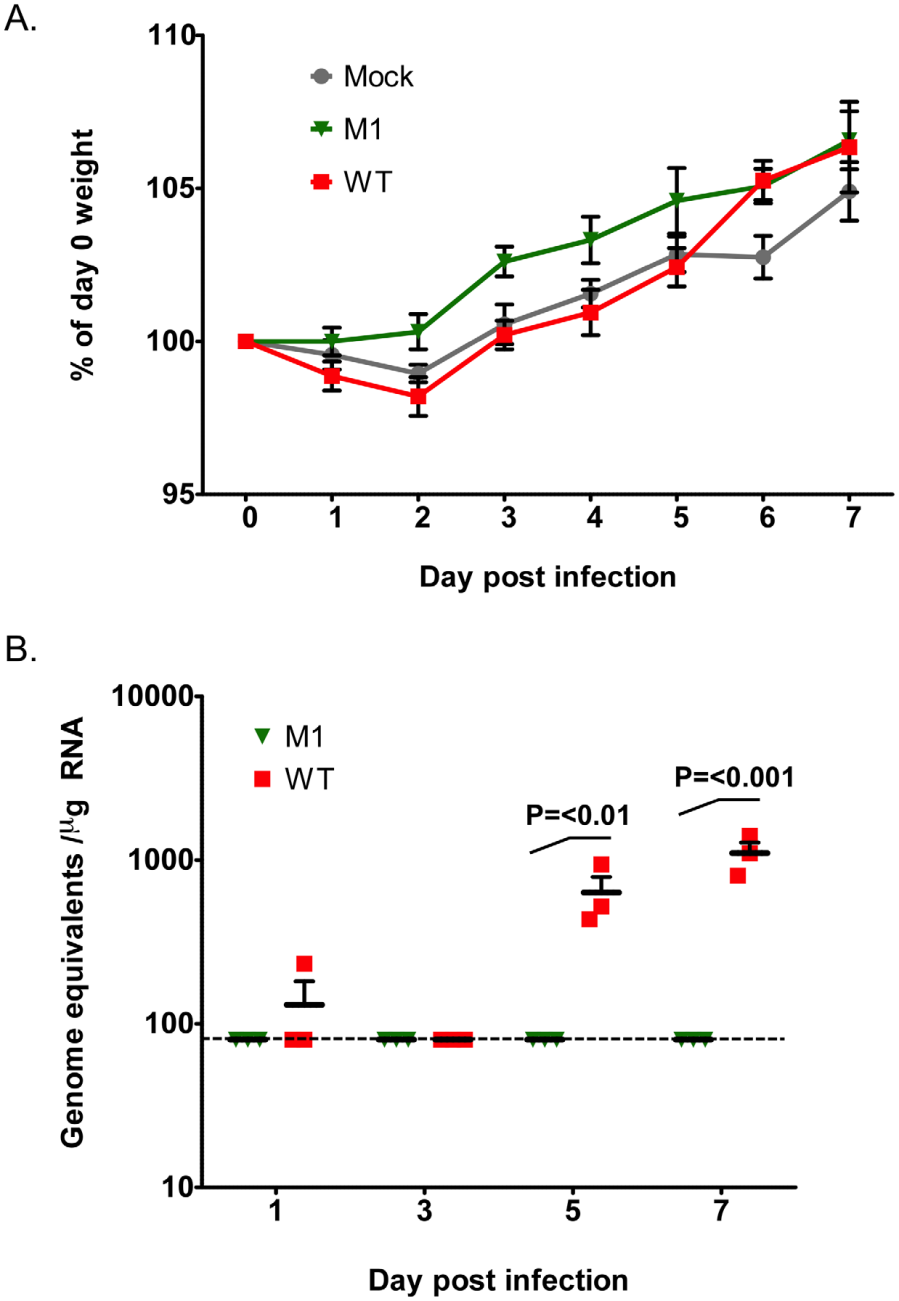
VF1 expression is required for virus efficient replication *in vivo*. Age and sex matched C57BL/6 mice were inoculated by oral gavage with 1×10^7^ TCID_50_ of low passage, sequence verified, wild-type (WT) or VF1 knockout (M1) viruses. Body weight was measured on a daily basis and expressed as a percentage of the weight on day 0 prior to inoculation. The mean weight and the standard error are plotted as a line graph covering the duration of the experiment (A). Quantitative real-time RT-PCR quantification of viral genome copies in the mesenteric lymph node isolated from mice at various times post inoculation (B). The mean and standard error are plotted. Statistical analysis was performed using two-way ANOVA and Bonferroni post tests (WT versus M1). The blue line represents the detection limit as determined by the sensitivity of qPCR to detect standards, equating to 80 copies per µg of RNA. Samples that were below the detection limit were set as 80 copies per µg of RNA.

The observed restoration of VF1 expression in the M1 and M10 viruses after repeated low multiplicity, multi-cycle replication in cell culture indicates that VF1 expression, although not essential for virus replication, confers some benefit to MNV-1 replication in cell culture. To examine if VF1 contributed to virus replication *in vivo* we examined replication in immunocompetent C57BL/6 mice. Whilst the isolate of MNV-1 used in this study and the only strain for which a reverse genetics system has been developed (CW1), is attenuated in the presence of a competent innate immune response [6], [42], low level virus replication in some tissues can be observed. Mice were inoculated with a high dose of low passage, sequenced verified WT and VF1 knockout viruses (M1) and the effect on body weight examined. As previously reported, no significant effect of MNV infection on weight loss was observed in this genetic background (Figure 7A). We also failed to detect robust levels of viral RNA in the small intestine, feces and spleen over the course of the experiment (data not shown). In contrast however, modest but significant levels of viral RNA were readily detected in the mesenteric lymph nodes of animals infected with WT MNV-1 at days 5 and 7 post infection (Figure 7B). In contrast, viral RNA was not detected in animals inoculated with the VF1 knockout virus M1. These data suggest that VF1 contributes to virus replication *in vivo* although virulence *per se* was not evident in this model even for the WT virus.

### VF1 is not required for replication of the virulent MNV-1 strain *in vitro*

To examine if VF1 contributes to MNV virulence, the robust STAT1 -/- mouse model for MNV was utilized. However, in order to undertake these studies it was first necessary to generate VF1 mutant viruses in a cDNA backbone virulent in STAT1-/- mice. We have previously demonstrated that the single nucleotide mutation A5941G, changing glutamate 296 to lysine in the major capsid protein VP1, was sufficient to restore virulence to the tissue culture adapted strain of MNV-1, which is attenuated in STAT1-/- mice [10]. The VF1 mutation M1 was generated in an MNV-1 cDNA clone bearing two mutations (G2151A and A5941G) as this sequence more faithfully represents the consensus sequence in viruses isolated from infected STAT1-/- mice [6,referred to as CW1.P1 in 10]. Initial analysis of the levels of virus obtained after reverse genetics recovery of the VF1 mutant virus M1 in the virulent backbone (referred to herein as M1-v), demonstrated identical levels to the wild-type virulent virus (WT-v) of approximately 1-5×10^3^ TCID_50_/ml (data not shown). This suggests that, as observed in the attenuated background, VF1 expression is not essential for MNV-1 replication in the STAT1-/- virulent backbone. To further verify this, multi-cycle growth kinetics analysis of low passage, sequence verified, WT-v and M1-v viruses in RAW264.7 cells was performed confirming equivalent growth kinetics (data not shown).

### MNV-1 viruses lacking VF1 are partially attenuated in STAT1-/- mice

**Figure 8:**
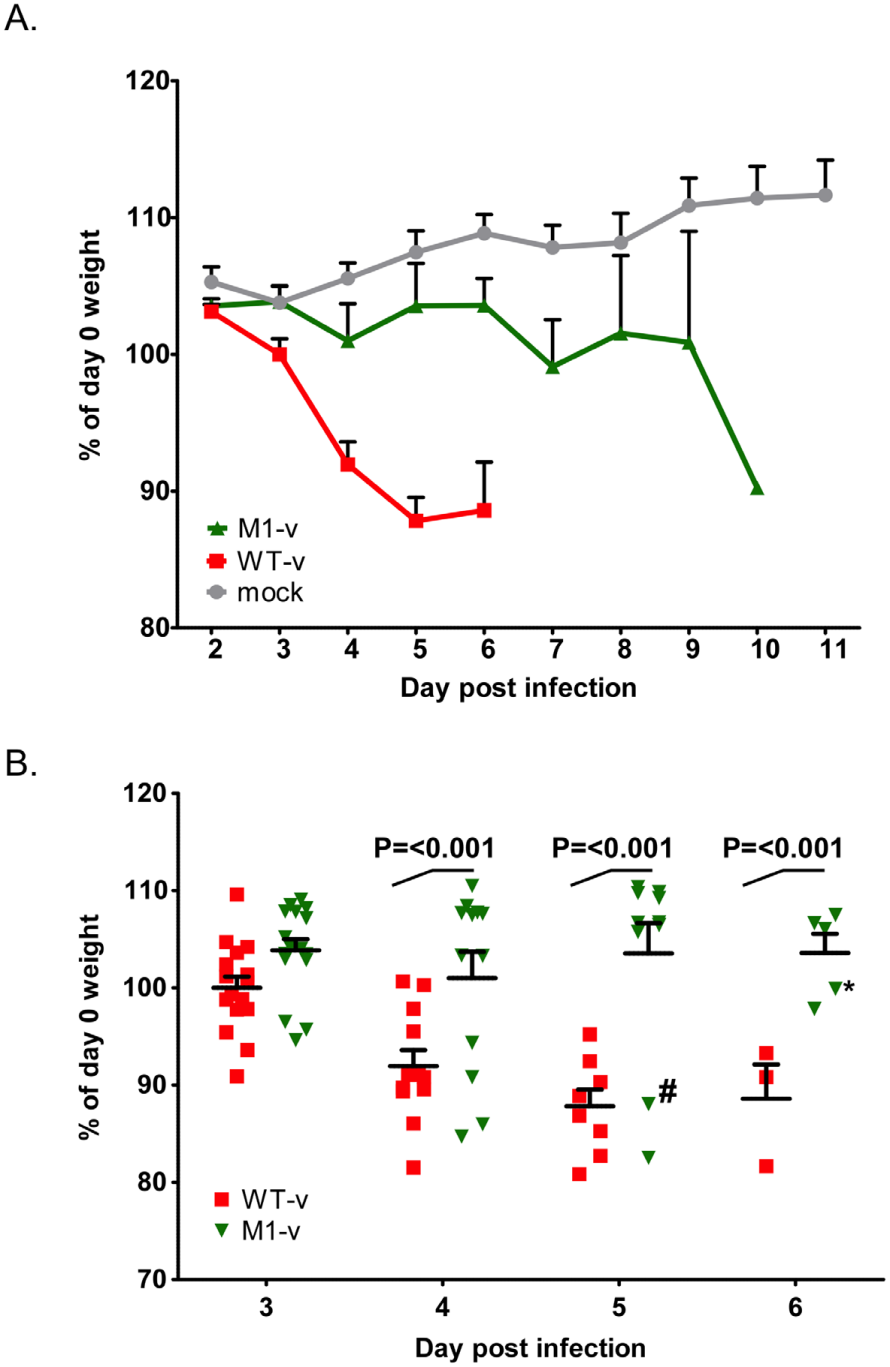
VF1 plays a role in viral virulence. Age and sex matched STAT1-/- mice were inoculated by oral gavage with 1000 TCID_50_ of low passage, sequence verified, wild-type (WT-v) or VF1 knockout (M1-v) viruses generated using a virulent backbone cDNA construct. As a measure of the severity of clinical disease, body weight was measured on a daily basis and expressed as percentage of the weight on day 0 prior to inoculation. Mock infected animals were orally inoculated with a control lysate preparation, generated as described in the materials and methods. For clarity the mean weight and the standard error are plotted as both a line graph covering the duration of the experiment (A) and as individual animal weights during days 3-6 (B). Statistical analysis was performed using two-way ANOVA with Bonferroni post-tests (WT-v versus M1-v). The hash and asterisk highlight animal number 776 and 751 used in the sequence analysis on days 5 and 7 respectively as described in the text. Note that animal 751 was removed from the study on day 7 (not shown on the plot) due to humane endpoints.

The ability of WT-v and M1-v viruses to infect and cause disease in the STAT1-/- mouse model was then examined by oral infection of age and sex matched mice. Oral inoculation of STAT1-/- mice with 1000 TCID_50_ units of low passage, sequence verified, wild-type virulent MNV-1 derived from cDNA (WT-v) resulted in the appearance of clinical signs (sunken eyes, reduced appetite, hunched inactivity and piloerection) as early as three days post inoculation. This was followed by a rapid and statistically significant (P<0.001) weight loss, when compared to animals inoculated with mock RAW264.7 cell lysate (day 4 onwards), and the development of more severe clinical signs culminating in significant weight loss (Figure 8). All WT-v infected mice succumbed to infection or were euthanized (because of disease severity limits being surpassed) by day 7 post infection. In stark contrast mice inoculated with the VF1 mutant virus (M1-v) showed a delayed onset of clinical signs. A statistically significant weight loss, compared to the mock-inoculated control group, was not observed until 6 days post infection (P<0.05, Figure 8). Of note, although the onset of M1-v associated disease was significantly delayed, all M1-v infected animals eventually succumbed to the infection or surpassed the severity limits of our trial. Experiments performed using a 10 fold higher dose (10,000 TCID_50_) also demonstrated that M1-v inoculated mice displayed a delayed onset of clinical signs including a statistically significant variation in body weight loss (two-way ANOVA). However this variation was markedly less than that observed at the lower infectious dose of 1000 TCID_50_ (Figure S2).

### Viral RNA replication is reduced in animals infected with the M1-v VF1 knockout virus

**Figure 9:**
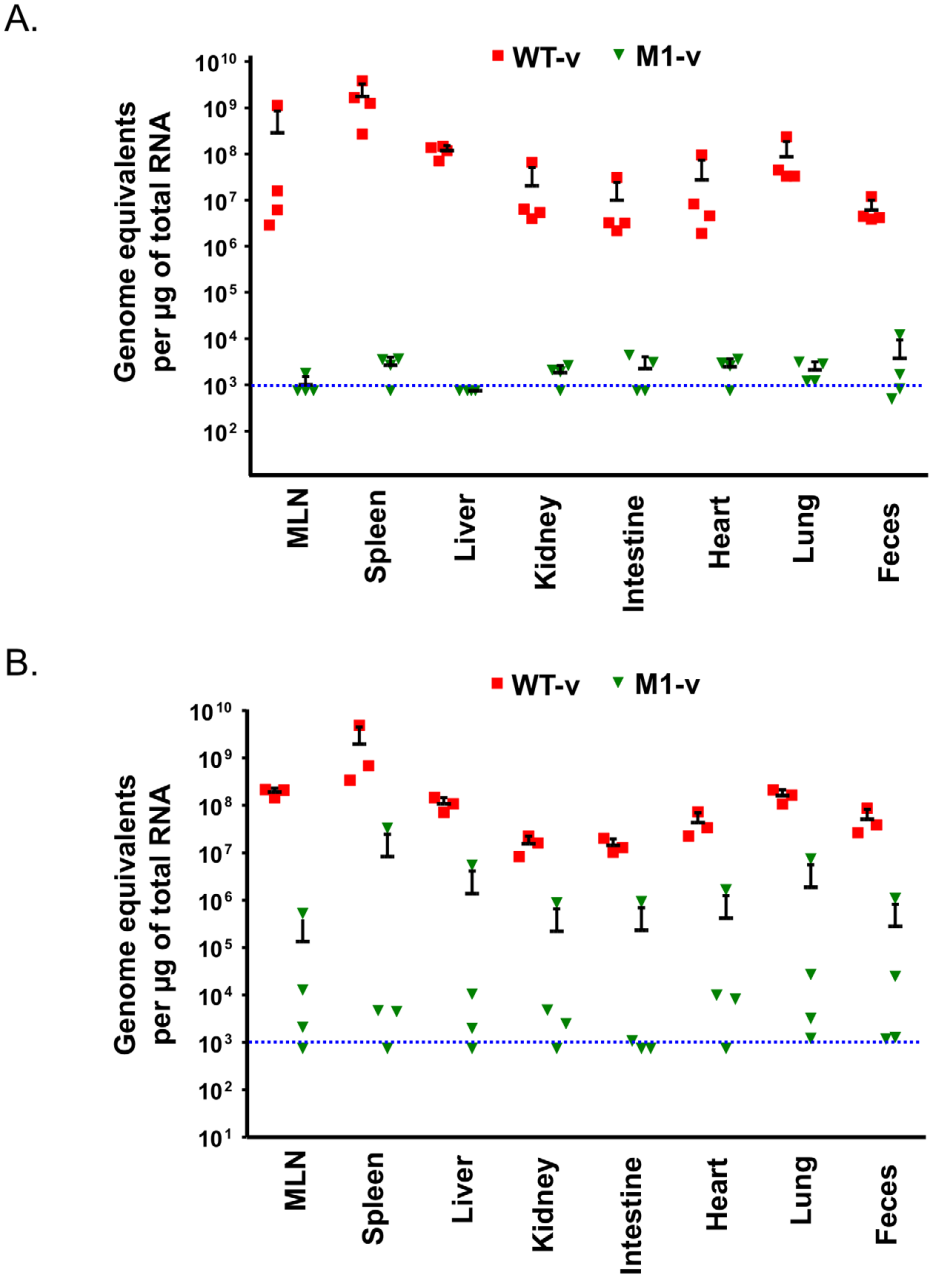
Viral RNA replication *in vivo* is reduced as a result of the loss of VF1 expression. Quantitative real-time RT-PCR quantification of viral genome copies in various tissues isolated at 3 (A) or 5 (B) days post infections with either wild type (WT-v) or VF1 knockout (M1-v). At various times post inoculation, tissue samples were isolated and RNA extracted. The genome equivalent per µg of RNA was then determined by comparison to a standard curve generated from *in vitro* transcribed RNA. The blue line indicates the limit of the detection, based on the ability to detect viral RNA specifically in RNA samples prepared from mock infected animals. Each RNA sample was analyzed at least in duplicate and the mean taken. The mean of each group and standard error is plotted. MLN refers to the mesenteric lymph nodes.

To examine if the distribution of virus replication differed between animals inoculated with WT-v or M1-v viruses, viral genome copies were quantified in various tissues at 3 and 5 days post infection by quantitative real-time reverse transcription PCR (qRT-PCR, Figure 9). Whilst >10^6^ genome equivalents (gEq) per µg of total RNA could be readily detected in samples from mice infected with WT-v 3 days post infection, viral genome levels in M1-v infected animals were typically 10^4^–10^5^ fold lower (Figure 9A). For example, average levels in the spleen for WT-v infected animals were 1.7×10^9^ gEq/µg of total RNA whereas M1-v inoculated animals showed an average of 2.6×10^4^ qEq/µg of total RNA. Increased viral RNA replication was detected in all WT-v infected mice at day 5 post infection but only in a subset of the M1-v infected mice (Figure 9B). This subset correlated with those animals that had developed more significant clinical signs and had lost body weight at day 5 post infection.

### M1-v infected mice show reduced histopathology at day 5 post infection

**Figure 10:**
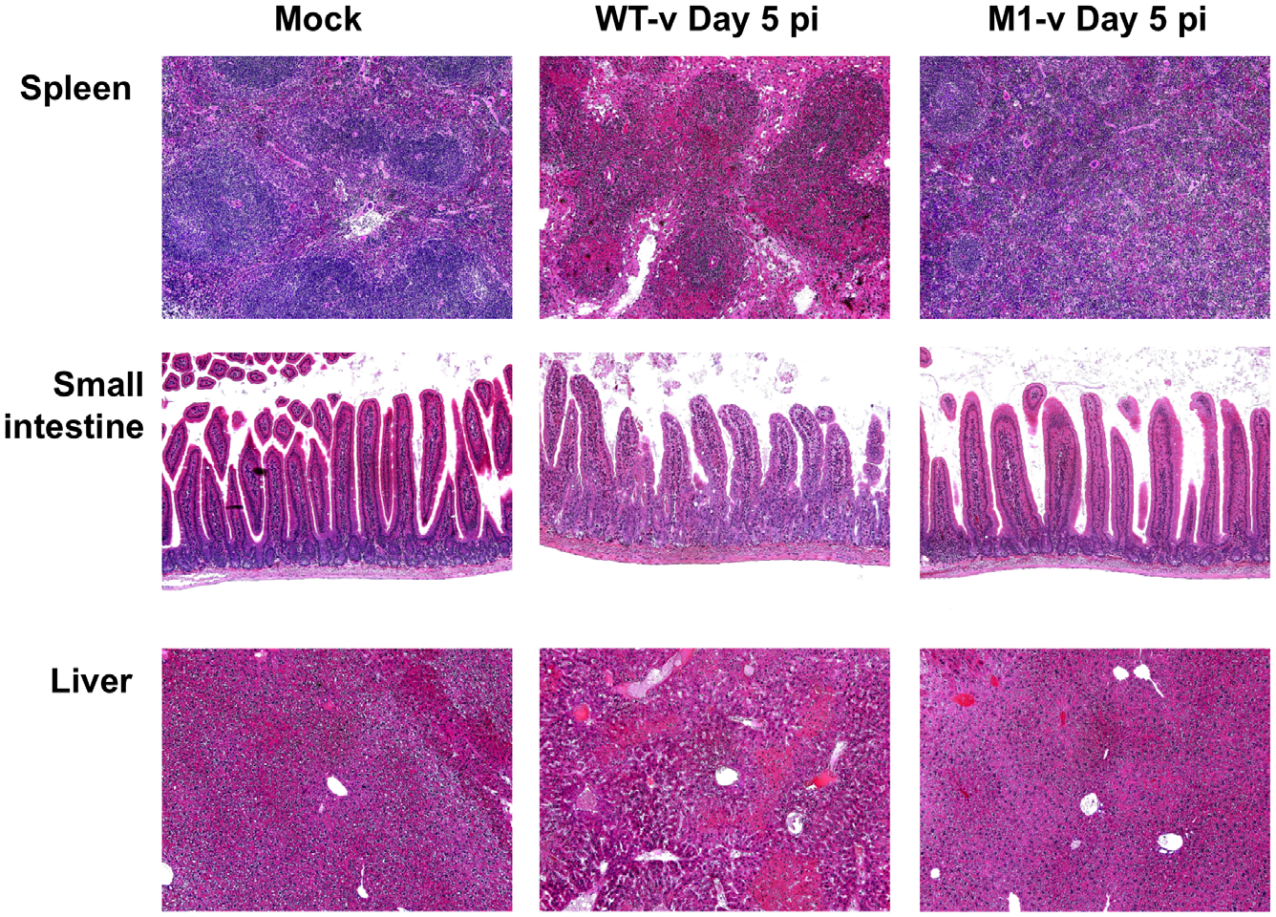
Pathology is markedly reduced in the absence of VF1 expression. Age and sex matched STAT1-/- mice were inoculated by oral gavage with 1000 TCID_50_ of low passage, sequence verified, wild-type (WT-v) or VF1 knockout (M1-v) viruses generated using a virulent backbone cDNA construct. Necropsies were performed 5 days post infection. Spleen, small intestine and liver tissues were fixed in Bouin's solution, embedded in paraffin wax and sections stained with haematoxylin and eosin.

Tissue samples from the spleen, small intestine and liver of mock, WT-v and M1-v infected mice were harvested at day 5 post infection for histopathological analysis on hematoxylin and eosin stained sections (Figure 10). Tissues from mock-infected mice were relatively normal for STAT1-/- mice (Figure 10). In contrast the WT-v infected tissues demonstrated reduced cellularity in spleen and liver, with foci of marked necrosis and apoptosis. Necrosis was evidenced by eosinophilia (dead cells staining bright pink) with pyknosis (nuclear condensation) and karryorrhexis (pyknotic nuclei become fragmented into several particles). Apoptosis was evidenced by cell rounding, a shrunken nucleus and in some cases cell fragmentation with some of the fragments containing apoptotic bodies. Blunting of the intestinal villi, as determined by measuring the villous height on the digital images taken at the same magnification and a comparison carried out on the mean of 8 villi, was apparent only in sections of the small intestine from animals infected with WT-v (Figure 10). Although the spleen from animals infected with M1-v appeared activated with partial paracortical hyperplasia, it was otherwise normal with little evidence of the necrosis or apoptosis evident in WT-v tissues (Figure 10). The liver revealed a partial loss of cellularity; however, evidence of apoptosis was again absent (Figure 10). In conclusion the lack of substantial pathology in M1-v infected mice at 5 days post infection correlated with our previous observations for differential viral RNA replication and weight loss in WT-v and M1-v infected mice.

### The M1-v virus does not revert during infection of STAT1-/- mice

The delayed virulence of VF1 knockout viruses in STAT1-/- mice was suggestive of reversion during replication *in vivo*. To examine this possibility further, sequence analysis of the viral population from all tissues in two animals displaying the highest degree of clinical signs (and viral genomes determined by qRT-PCR) was undertaken. The animals analyzed are highlighted in Figure 8B with a hash and asterisk indicating animals culled on days 5 and 7 respective. The animal culled on day 5 displayed similar disease onset to that of WT-v inoculated animals and had lost ∼12% of its initial body weight. The animal culled on day 7 for sequence analysis was removed from the study due to human end points being exceeded and had lost ∼7.6% of its initial body weight. Consensus sequence analysis, which under the conditions used could reproducibly detect reversion when ∼25% of the population had restored VF1, failed to detect any reversion in any of the tissues (Figure S3). In addition, tissues/samples containing the highest viral loads (4×10^6^ to 4×10^7^ copies per µg of RNA), namely the spleen and feces from the day 5 animal and the spleen from the day 7 animal, were PCR amplified and 10 individual clones sequenced. Of the 30 clones sequenced, none contained a mutation in VF1 that would lead to restoration of VF1 expression, further confirming the lack of detectable reversion upon replication a single pass in Stat1-/- mice *in vivo* (data not shown).

## Discussion

Studies on numerous RNA viruses have identified the use of overlapping open reading frames to maximize the coding capacity of their small RNA genomes [2]. These ORFs and their proteins typically play accessory roles in the viral life cycle such as modulating the host immune response to infection [43]-[45]. Frequently they dispensable for viral replication in immortalized cell lines; however, it is the *in vivo* setting that the true requirement for these proteins in the viral life cycle is apparent. This study indicates that MNV should now be added to the list of viruses that have adopted this strategy to maximize the coding potential of their genome.

Initial bioinformatic investigation of MNV complete genome sequences identified a conserved ORF overlapping with ORF2 (Figure 1), potentially translated from the sgRNA produced during infection. Traditionally the sgRNA is thought to encode only the major and minor capsid proteins, VP1 and VP2. However suppression of variability in this region and conservation of the alternate ORF was shown to be absolute in all available MNV sequences (Table 1). Although the resultant full length protein, VF1, was recalcitrant to high level expression and poorly immunogenic, polyclonal antibody specific to this protein was generated and used to confirm expression during infection (Figure 2). The efficient translation of this protein was confirmed by immmunoprecipitation following translation of the sgRNA *in vitro* (Figure 2).

This is the first confirmation of the expression of an internal open reading frame protein for any member of the *Caliciviridae*. The internal open reading frame encoding VF1 can be found in all currently published MNV genome sequences, highlighting the requirement for this feature in the MNV genome. The evolutionary conservation of ORF4 coding sequences and the marked suppression of sequence variability localising specifically to the area of overlap (Figure 1C) provides evidence independent of the *in vitro* data for a functional requirement to maintain an intact ORF4 reading frame. As indicated by the analysis of leucine and other synonymous codon usage, this selection pressure was sufficiently strong to drive unfavoured codons into the ORF4 coding sequence (Figure 1C), a feature exploited by MLOGD [33] to detect regions of multiple coding.

There were considerable similarities in the arrangement and translation contexts of the ORF2 and ORF4 genes of MNV with documented regions of multiple coding in other viruses. The MNV ORF4 has an initiating AUG triplet at position 5069 in a strong Kozak context (G at -3 and +4 [46]. It is positioned 13 bases downstream from the first AUG triplet of ORF2 (weak context; U at -3, A at +4) and 7 from the second (adequate context; A at -3 and +4). This arrangement of initiating codons in the MNV sgRNA transcripts is similar to viral [47] and eukaryotic [48] dicistronic mRNAs in which alternative weak context initiating codons around an initiating codon in a strong context (ORF4 in MNV) can be accessed by random forwards and backwards movements of the ribosome from its initial binding site, termed "leaky scanning". In this case, this would require a backwards movement to the second AUG triplet of ORF2, remarkably similar to the documented dicistronic expression of p206 (strong context) and p69 (weak context 7 bases upstream) from genomic RNA of turnip yellow mosaic virus [47]. This hypothesis is supported by the observation that noroviruses that lack ORF4 (genogroups 1–4) show a strong Kozak context around the second AUG triplet in ORF2 (A at -3, G at +4). The evolutionarily conserved nucleotide difference at position 5065 (+4) between MNV (A) and other noroviruses (G) may thus play a key enabling role in the hypothesised dicistronic expression of ORF4 and ORF2 by MNV.

The juxtapositioning of ORF4 at the start of the sgRNA gives an indication of the additional evolutionary constraints that this ORF, and the respective protein, must be under. This region of the genome contains multiple conserved *cis*-acting RNA elements that play an important role in the viral life cycle (unpublished observations). It is important to note however that the single nucleotide mutation introduced in this region to generate the M1 virus, did not affect the structure of these RNA elements as we have determined biochemically that nucleotide 5118 is positioned within a single-stranded region (data not shown). ORF4 also overlaps with the region of ORF2 that encodes the shell (S) domain of the major capsid protein, VP1. Dimerization of the S domain is thought to be integral for the development of the icosahedral core of the virus particle and is consequently the most conserved domain in VP1. As the S domain is buried inside the virus particle, it is unlikely to be under strong antibody selection pressure, unlike the more variable C terminus of VP1 which contains the protruding (P) domain. The contribution of all these factors is likely responsible for the low divergence observed between MNV VF1 sequences (Table 1).

Within the norovirus genus, ORF4/VF1 appears to be a feature unique to MNV as other noroviruses appear not to encode an equivalent open reading frame. The human noroviruses, which represent a significant cause of viral gastroenteritis in man, do not share the extensive suppression of synonymous site variability at the start of ORF2 that first indicated the presence of ORF4 (Figure 1C) [13]. Direct analysis of the available human norovirus sequences confirms that no such ORF exists (data not shown). A broader analysis of the *Caliciviridae* family indicates the presence of an equivalent open reading frame in some strains of human sapoviruses (data not shown) [49]. Although there is low sequence homology between the respective proteins (25% similarity, 18% identity) the presence of this alternative ORF indicates a potential conserved mechanism for maximizing coding potential. It is also possible that a common ancestor of all caliciviruses possessed an equivalent ORF, which has subsequently been lost in the case of the majority of caliciviruses. Although human noroviruses, as well as other members of the *Caliciviridae*, lack an equivalent ORF4 within the VP1 coding region of the sgRNA, we cannot at this point rule out functional duplication i.e. that the functions of MNV VF1 have been duplicated in human noroviruses by one of the other viral proteins. Further studies are therefore warranted to determine if human noroviruses and other members of the *Caliciviridae* also possess the ability to modulate the innate immune response.

The role of the VF1 protein in MNV-1 replication was examined using the permissive macrophage RAW264.7 cell line and the reverse genetics system developed previously in our laboratory [22]. A series of VF1 truncations, generated by inserting stop codons into ORF4, which importantly left the VP1 coding sequence unaltered, confirmed this protein was a classical viral accessory protein not required for replication. However, repetitive multicycle, low multiplicity of infection passage in the permissive RAW264.7 cell line resulted in a phenotypic reversion for the more severe truncations (M1 and M10) (Figure 3), demonstrating that VF1 expression and function benefits virus replication in cell culture. Whilst this observation was reproducible, it was clear that rapid phenotypic reversion did not occur as virus stocks generated by reverse genetics and subsequently amplified in cell culture by a single passage maintained the introduced mutations. The phenotypic reversion observed was the likely result of the multicycle nature of the infections as very low multiplicity of infections were used, resulting in multiple rounds of virus replication to occur in each pass in cell culture.

As the ‘powerhouses’ of the eukaryotic cell, viruses often modulate the function of mitochondria to maintain an intracellular environment beneficial for viral replication. The mitochondrial localization of VF1 (Figure 4) together with its apparent modulation of innate immune signaling and apoptosis (Figure 5 and 6) indicates that this protein may function, like so many other viral proteins, to facilitate viral replication and antagonize anti-viral mechanisms adopted by the cell. The beneficial functions of VF1 expression, e.g. delayed apoptosis and innate immune responses in infected cells, are apparent since analysis of VP1 protein levels produced during infection are clearly reduced at the later stages of infection in the absence of VF1 (Figure 6B). Surprisingly this does not appear to affect the final yield of virus (Figure 3D) or the levels of viral RNA produced during replication in RAW264.7 cells (data not shown). The RAW264.7 murine macrophage cell line is extremely permissive to infection and it is likely that the total pool of available VP1 protein in the M1 infected cells later in infection does not limit virion production. Of relevance is our observation that the IRF3 modulated gene ISG54, also known as IFIT2 or p54, is significantly upregulated in cells infected with a virus lacking VF1 (Figure 5A). The ISG54 protein (p54 or IFIT2) functions to repress cellular translation by binding and inhibiting the cellular eIF3c protein [50]. Previous work has indicated that norovirus translation initiation may require the eIF3 complex via a direct interaction of VPg [51]. The expression of the MNV VF1 protein may therefore delay or block the ISG54 mediated inhibition of cellular and viral VPg-dependent translation by preventing the induction of ISG54 mRNA at the point of mitochondrial mediated activation of the innate immune response [51]–[53]. ISG54 expression has also been linked to apoptosis induced via the mitochondrial pathway [54], again in agreement with our observed increase in apoptosis in the absence of VF1 expression (Figure 6). It is possible therefore that the increased apoptosis observed during virus replication in the absence of VF1 expression is as a result of the increased induction of the innate immune response.

It is well established that MNV is sensitive to an effective innate immune response: type I and II interferon are known to inhibit viral translation [20], a fact supported by the observed sensitivity of STAT1-/- mice to infection [6]. The role of STAT1 mediated, interferon-based, innate immune signaling in combating MNV-1 infection has been well characterized to prevent the progression of MNV-1 infection and dissemination to peripheral tissues [42]. We also observed this effect in our studies with immunocompetent C56BL/6 mice as only low levels of viral RNA were detected in the MLN (Figure 7). Previous work has also highlighted that at least part of the innate sensing of MNV infection by the host cell can be attributed to the MDA5 protein [17]. When activated, MDA5 signals the detection of viral RNA through the mitochondrial antiviral signaling (MAVS) adapter protein (also known as IPS-1, VISA or Cardif) embedded in the outer membrane of this organelle [36]–[38]. One downstream consequence of these signaling events at the mitochondria is the dimerisation and subsequent nuclear shuttling of IRF3. This activation of IRF3 results in the trans-activation of genes responsible for combating viral infection including interferon beta. In our studies, the regulation of genes specifically stimulated by virus infection was monitored by qPCR and ELISA and shown to be elevated in cells infected with a virus lacking the VF1 accessory protein (Figure 5A and Figure 5B). This potential role for VF1 as an antagonist of the innate immune response was investigated using co-expression studies with various auto-stimulatory components of this pathway which, after transient over-expression, are known to trigger interferon production (such as RIG-I, MAVS) (Figure 5C). In this instance, VF1 was shown to reduce the expression of a reporter protein under the direct transcriptional control of the interferon beta promoter. This occurred at the level of, or subsequent to TBK1 activation, since its stimulation of the IFN promoter was also inhibited by VF1 expression. The mechanism of action of VF1 therefore potentially involves modulation of the interaction of TBK1 with IRF3, or directly acts on IRF3 itself. Inhibition or degradation of IRF-3 is a frequent target of viral evasion strategies among both RNA and DNA viruses, including the Npro protein of pestviruses [55], [56], the P protein of rabies virus [57] and the G1 protein of hantaviruses [58]. Mitochondria also serve as a platform for the activation of IRF3 via TBK1 as the mitochondrial import protein Tom70, interacts with MAVS upon RNA virus infection and subsequently recruits the TBK1-IRF3 complex via Hsp90 [59]. The interaction of Tom70 with cytosolic chaperone Hsp90, which is itself constitutively associated with TBK1 and IRF3, plays a critical role in the activation of IRF3. Therefore another possible mechanism of VF1 function may be via the modification of the mitochondrial activation pathway or the formation of the MAVS-Tom70-Hsp90 complex.

The role of VF1 during *in vivo* infection was initially examined in a wild type immunocompetent mouse background. However, the strain of MNV-1 used during these studies and the only strain for which a reverse genetics system is currently available, namely CW1, is attenuated in this model, failing to produce any obvious clinical signs (Figure 7). It is worth noting however that this variant of MNV-1, although attenuated in STAT1-/- mice due to a glutamate at position 296 in the major capsid protein VP1 [10], is actually more representative MNV isolates from immunocompetent mice as they typically also contain glutamate at position 296 [32]. Using this variant of MNV-1 CW1 in this genetic background we observed viral RNA in the mesenteric lymph node at 5 and 7 days post infection with WT MNV-1 but not when animals were infected with a virus lacking VF1. These data add further strength to our hypothesis that VF1 expression is beneficial to virus replication as it delays the innate immune response and as a consequence, virus induced apoptosis. To examine the role of VF1 in MNV-1 virulence, the M1 VF1 truncation was first engineered into the virulent MNV-1 backbone, previously described by our laboratory [10]. This virus, which represents the closest available progenitor of the original isolate of MNV identified in 2003, causes a lethal infection in STAT1-/- mice [6]. Typically, one might expect to observe a restoration of virulence after infection of STAT1-/- mice with a virus that lacks an interferon antagonist, in this case VF1. One of the best-studied examples of this is in influenza virus where the lack of NS1 has no effect on virulence in STAT1-/- mice but in immunocompetent mice a deletion of NS1 results in attenuation [60]. However, In the case of the MNV-1 studies undertaken here, the *in vivo* analyses are complicated by the already attenuated nature of the strain used (CW1) in an immunocompetent host. Infection of STAT1-/- mice with either 10^4^ or 10^3^ TCID_50_ of WT-v or M1v demonstrated that MNV-1 lacking VF1 was partially attenuated in this system exhibiting delayed replication kinetics in the murine host (Figure 7, 8, 9 and 10). This manifested as a delay in both the onset of typical MNV-1 disease and the associated presentation of symptoms (weight loss, piloerection, anorexia, eye discharge that subsequently develop to ataxia, moribundity and death). Quantification of the viral RNA genomes in infected tissues at days 3 and 5 post infection, as well as the gross differences in MNV-1 related pathology at day 5 are testament to the debilitated replicative ability of this virus *in vivo* even in the absence of STAT1. The exact mechanism of this attenuation is unclear since all the inoculated animals (M1-v or WT-v) eventually developed disease and either succumbed to infection or had to be euthanized due to the established humane end points being surpassed. Detailed analysis of the function of VF1 in the avoidance of the innate immune response *in vivo* is likely to require the development of a reverse genetics system for a MNV variant capable of infecting immunocompetent mice. In the absence of this however, we are able to offer at least one possible explanation as to why we observed clinical disease in the STAT1-/- model even in the absence of VF1. In STAT1-/- mice, the absence of an intact STAT1-dependent interferon response pathway prevents the generation of robust autocrine and paracrine interferon responses. There are however many examples of virus infection leading to the induction of host genes classically defined as interferon stimulated genes (ISGs) in the absence of interferon and/or STAT1 mediated signalling; examples include LCMV [61] where the induction of ISG-49, ISG-54, and ISG-56 was observed in the absence of STAT1, and also HSV-1 which elicits an IRF3-dependent, but IFN-independent cellular antiviral response [62]–[64]. Direct IRF3 mediated responses are also known to protect against West Nile virus infection in both interferon dependent and independent mechanisms [65]. In addition, recent studies have highlighted that STAT2-mediated signalling may stimulate the expression of a subset of ISGs in the absence of STAT1 [66]. Therefore we would propose that during our studies in the STAT1-/- mouse model, it is likely that infection with the virus lacking VF1 leads to the induction of a subset of ISGs during virus replication at the primary site of infection, either directly via an unknown mechanism, or via STAT2. This limited response may slow virus replication, resulting in the delayed virus replication at the initial site of infection, reduced virus production and delayed onset of disease, all consistent with our observations. We would predict however that this limited response is not sufficient to clear virus after multiple rounds of infection. Our preliminary analysis would confirm that infection of STAT1-/- mice with virulent WT MNV-1, can result in the induction of ISGs, even in the absence of STAT1, as we observed increased levels of CXCL10 and ISG54 at 3 days post infection (Figure S4). The mechanism of ISG induction in the absence of STAT1-mediated signalling and how VF1 contributes to virulence in the absence of STAT1 will require further studies.

Expression of accessory proteins from alternate open reading frames can be found in many RNA viruses, many of which parallel the ability of VF1 to antagonize the innate immune system (discussed in more detail below). Many negative strand RNA viruses from the *Paramyxovirus* genus encode alternative proteins from internal ORFs in the phosphoprotein mRNA. These proteins, denoted C, play multiple roles in the viral life cycle that include the facilitation of RNA replication and control of the innate-immune response [67]. Sendai and measles virus mutants lacking C are viable in tissue culture but partially attenuated *in vivo* [68], [69]. This inability to replicate as efficiently as the wild-type virus *in vivo* is comparable to the observed results in this study for MNV-1 VF1 (Figure 7, 8, 9 and 10).

The influenza protein PB1-F2, another viral protein produced from an alternate open reading frame, provides additional evidence for the role of these accessory proteins in disease [43], [44], [70]. PB1-F2 is a recently discovered virulence factor, encoded by the PB1 gene segment, which interacts with mitochondria and stimulates apoptosis by facilitating cytochrome c release via interactions with ANT3 and VDAC1 [71], [72]. In addition PB1-F2 has been shown to affect influenza polymerase activity in the nucleus, modulate interferon responses during infection and, interestingly, to exacerbate secondary bacterial infections *in vivo* [71]. Despite the mitochondrial interactions of PB1-F2, it is unlikely that VF1 functions in an analogous manner since apoptosis was exacerbated in cells infected with a virus lacking VF1 (Figure 6). A recent report demonstrates a link between the innate immune response and apoptosis suggesting that both MAVS and IRF3 may play direct roles in stimulating apoptosis [73], [74]. ISG54 expression is also known to induce apoptosis [54]. It is possible that this exacerbation of apoptosis, in the absence of VF1, is a by-product of enhanced activation of the innate immune response in cells infected with the M1 virus (Figure 5 and 6) or an as yet uncharacterized direct or indirect modification of MAVS function by VF1. Interestingly, the HCV NS3/4A [75] and SARS-CoV NSP15 protein have been shown to be inhibitors of MAVS mediated apoptosis, identifying these proteins as potential orthologs of VF1 [73].

Other potential ARFP proteins have also recently been shown to associate with the mitochondria [76] as has the L* protein of Theiler's murine encephalomyelitis virus (TMEV) [77]. L* is only encoded by the TO subgroup of TMEV viruses where it is required for growth in macrophages and has been implicated in the establishment of persistence and the demyelination associated with TMEV [78]. This macrophage specific requirement for L* is paralleled, albeit to a lesser degree, for MNV VF1. In our study the M1 and M10 knockout viruses phenotypically reverted upon repeated, low multiplicity, multy cycle replication in the RAW264.7 murine macrophage cell line, highlighting that VF1 expression and function confers some benefit to MNV growth in this cell line (Figure 3E and 3F). This benefit is the likely combination of the observed increase in apoptosis and innate immune signalling observed in cells infected with a virus lacking the VF1 protein.

Numerous other examples of viral proteins that interact with the mitochondria during infection include the HBV X gene protein whose interactions are thought to stimulate apoptosis and play a role in the development of cancer in affected individuals [79], the HCMV UL37 protein which is thought to modulate Ca^2+^ signaling and apoptosis at the mitochondrial ER synapse [80], as well as three hepatitis virus proteases that have been shown to cleave MAVS (Hepatitis A, B and C), the adapter for RIG1 and MDA5 signaling, thereby antagonizing the innate immune response to infection [81]. Given the critical role that mitochondria play in cellular responses to infection and stress e.g. innate immune signaling, calcium homeostasis and regulation of apoptosis, it is maybe not surprising that so many viruses target this organelle.

Our studies have demonstrated that MNV-1 encodes a novel virulence factor from an alternate open reading frame in the sgRNA. This protein localizes to the mitochondria during infection and apparently inhibits the signaling events that take place in and around this organelle during infection of the host cell. This appears to affect the downstream activation of genes regulated by mitochondrial arm of the innate immune response and also the development of apoptosis in response to infection. A mutant virus lacking the ability to express VF1 does not replicate as efficiently in immuncompetent or STAT1-/- mice, which manifests as a delayed onset in the development of disease. However this does not protect the mice from developing serious disease highlighting the sensitivity of this specific model. The role of VF1 in the establishment of persistent MNV infection as well as the exact nature of VF1 interaction with the mitochondria and the mechanism by which this interaction modulates the function of this organelle is the subject of continued research in our laboratory. The identification and preliminary characterization of the MNV-1 VF1 protein provides a unique perspective on this widely used model pathogen and may provide additional insights into the mechanism of norovirus evasion of the immune system.

## Materials and Methods

### Ethics statement

All of the STAT1-/- animals used in this study were maintained at an American Association of Laboratory Animal Care-accredited animal facility at UTSW Medical Center and the protocol was approved by the IACUC at UT Southwestern Medical Center (Permit number: 1151). Animal use adhered to applicable requirements such as the Animal Welfare Act (AWA), the Guide for the Care and Use of Laboratory Animals (Guide), the Public Health Service Policy, and the U.S. Government Principles Regarding the Care and Use of Animals. Studies involving C57BL/6 mice were performed at Imperial College London St Mary's Campus (PCD 70/2727) after ethical review by the Imperial College Ethical Review Panel and subsequent approval of the British Home Office (PPL 70/6838). All animal procedures and care in the UK conformed strictly to the United Kingdom Home Office Guidelines under The Animals (Scientific Procedures) Act 1986.

### Cell culture and cell lines

MNV-1 was propagated in the murine leukaemia macrophage cell line RAW264.7 using Dulbecco modified Eagle medium (DMEM) with 10% fetal calf serum (FCS), penicillin (100 U/ml), streptomycin (100 µg/ml) and 10 mM HEPES (pH7.6). COS7 and MEF cells were cultured in DMEM (with FCS and pen/step as above). Baby-hamster kidney cells expressing T7 DNA polymerase (BSRT7 cells) used during reverse genetics recovery of MNV-1 from cDNA clones, were obtained from Klaus Conzelmann (Ludwig-Maximilians-University Munich) [82] and cultured in DMEM (+FCS and pen/step as above) containing G418 at a concentration of 1 mg/ml. All cells were maintained at 37°C with 10% CO2.

### Antibodies

Repeated attempts to immunize rabbit with full length his-tagged MNV-1 VF1, purified from *E.coli*, failed to illicit a robust immune response. Therefore a modified immunization regime that used a combination of peptide immunization followed by booster injections with various recombinant proteins was required. Full details are available upon request, but briefly animals were immunized with the peptide (PGKLTKLTPGSSKIL), representing amino acids 42–56 of VF1 conjugated to KLH, then boosted with the same peptide. This was followed by two subsequent booster injections using full length recombinant his-tagged VF1 expressed in and purified from *E.coli.* One booster injection with amino acids 42–70 (PGKLTKLTPGSSKILSSAPLVSFPSRLET) fused to a Cherry/his tag (Cherry-VF1-his), expressed and purified from *E.coli* using the Cherry express system (Eurogentec) was also performed. Animals were subsequently boosted again with the primary peptide immunogen conjugated to KLH, followed by a final boost with Cherry-VF1-his. VF1 specific antiserum was then affinity purified from sera on a column generated using the initial peptide immunogen. Antisera to the MNV-1 VP2 protein, the product of ORF3, was generated by immunization of rabbits with full-length recombinant his-tagged protein expressed and purified from *E.coli*. Note that some batch-to-batch variation of the anti-VP2 antisera was observed resulting in minor differences in the staining intensity of background non-specific bands. In some cases antisera was pre-adsorbed by prior incubation with membranes on which uninfected samples has been run to remove the non-specific reactivity. Antisera to the MNV-1 VP1 protein was kindly supplied by Skip Virgin (Washington University in St Louis) and was used as previously described [6].

### ORF4 bioinformatic prediction

The following 28 full length genomic sequences were downloaded from GenBank and used for bioinformatic prediction of open reading frames: DQ223042, EU854589, EU004665, FJ446720, FJ446719, AB435514, EU004660, EU004672, EU004679, EU004681, EU004682, EU004674, EF531291, EU004673, EF531290, DQ911368, EU004683, EU004670, EU004668, EU004663, EU004671, EU004664, EU004677, EU004676, DQ223041, DQ223043, EU004678 and EU004680. Sequences were selected based on showing >1% sequence divergence from all other sequences and thus representing different MNV isolates.

Synonymous and amino acid variability for each ORF coding sequence were calculated using the program Sequence distance in the Simmonic sequence editor as previously described [13]. Variability at each position was averaged over 11 adjacent windows of 50 codons incrementing by 3 bases/window. MLOGD [33], [83] was used through the web interface (http://guinevere.otago.ac.nz/aef/MLOGD/). The MNV phylogeny used to generate the Pairs files was created by PHYLIP version 3.62 [84] using DNADIST (Jukes-Cantor corrected distances) and NEIGHBOR programs. Likelihood scores were calculated for the existence of ORF4 in addition to ORFs 1, 2 and 3 (gene positions 6-5066, 5056-6678 and 6681-7307 added as annotation).

### Mitochondrial isolation

2×10^7^ RAW264.7 cells were infected with MNV-1 at a MOI of 5 TCID_50_ per cell. After 12 h at 37°C, total cell lysates were prepared by washing in PBS and lysing directly into reducing SDS sample buffer. The mitochondria and cytosol of infected cells were separated using a mitochondria isolation kit for mammalian cells (Thermo scientific). The isolated mitochondria were directly suspended into reducing SDS sample buffer whilst due to the high volume the cytosolic fraction was concentrated using the UPPA-protein concentration reagent (G-Biosciences). Fractions were separated on a 15% SDS PAGE gel and analyzed by western blot using antibodies against VF1. A rabbit antibody against poly rC-binding protein1 and 2 (PCBP) acted as a control for the cytosolic fraction and the commercial goat anti-apoptosis inducing factor (AIF) antibody (D-20 from Santa Cruz Biotechnology) acted as a control for the mitochondrial fraction. VF1 and PCBP were detected using secondary HRPO conjugated anti-rabbit antibodies, whilst AIF was detected using a secondary HRPO conjugated donkey anti-goat antibody (Santa Cruz Biotechnology).

### Plasmid construction

VF1 mutant viruses were generated by the insertion of stop codons at various positions within ORF4, which disrupted VF1 production without affecting the amino acid coding sequence of the major capsid protein VP1. The mutations were generated in the previously described MNV-1 cDNA clone pT7:MNV 3’Rz [22] by PCR mutagenesis (primer details available upon request) using KOD hot start DNA polymerase (Novagen). The VF1 mutant virus M1 contains a stop codon through mutation of T to A at genome position 5118 and hence translation of VF1 terminates after 16 amino acids. The VF1 truncated virus M10 contains a T to A mutation at genome position 5364 terminating VF1 translation after 98 amino acids, and the truncated M20 contains a G to A mutation at genome position 5655 terminating VF1 translation after 195 amino acids.

The VF1 expression plasmid pcDNA3.1+MNV-1 VF1 was generated by cloning the VF1 encoding sequence into the expression plasmid pcDNA3.1+, which contains a CMV and T7 promoter (primer details available upon request). GFP fusions of VF1 were generated by cloning the VF1 encoding sequence into the GFP plasmids pEGFP-N1 and pEGFP-C1 (Clontech).

### Reverse genetics recovery of ORF4 mutant viruses

ORF4 mutant viruses were recovered using reverse genetics as previously described [22]. Briefly, BHK cells expressing T7 polymerase (BSRT-7) were infected with FPV expressing T7 polymerase and were subsequently transfected with the MNV-1 full length clones (pT7 :MNV 3’Rz) containing the VF1 mutations M1, M10 or M20 (described above). 24 h post transfection cells were frozen and the clarified lysates were used to generate passage 1 and 2 stocks by infecting RAW264.7 cells at low MOI and freezing 48 hours post infection. The virulent WT and VF1 knockout viruses used *in vivo* were generated by the same means, although the virus underwent only a single pass in tissue culture to prevent the appearance of the tissue culture adapted mutations at genome positions 2151 and 5941 as described previously [10]. Viral titres were determined by TCID_50_ titration in RAW264.7 cells. Prior to use, all viruses were sequenced to ensure they contained the relevant mutations.

### Growth kinetic analysis

RAW264.7 cells were seeded at 3.2×10^5^ cells per well in a 24-well plate and subsequently infected with the VF1 knockout (or truncated) viruses M1, M10 or M20 at an MOI of 0.01 TCID_50_ per cell. The assay was performed in triplicate for each virus. At given time points (0, 6, 12, 24, 48, 72 hours post-infection) the infections were frozen and upon thawing the viral titers were determined by TCID_50_. Protein samples over the given time course were also taken to analyze the kinetics of viral protein expression (data not shown).

### Analysis of ORF4 mutant virus stability

RAW264.7 cells were seeded at 3.75×10^6^ cells per well of a 6-well dish and were subsequently infected with ORF4 mutant viruses M1, M10 or M20 at a MOI of 0.01 TCID_50_ per cell. Note that the initial virus stock used for the infections were generated by reverse genetics recovery followed by a single passage in cell culture, so were effectively passage 1. The ORF4 region of the input viruses was sequenced prior to use. After 48 h the resulting ‘pass 1’ cultures were freeze-thawed and used to set up the subsequent low MOI (<0.01 TCID_50_ per cell) infections. Virus passage was continued for 5 cycles after which cells were infected at high MOI and RNA was isolated 12 h post infection. RT-PCR reactions were subsequently used to sequence the region of the genome encompassing ORF4 (primer details available upon request).

### Reporter assays of the effect of VF1 expression in the innate immune response

MEFs were seeded into 12 well dishes and transfected the following day using Lipofectamine 2000 (Invitrogen) with a reporter plasmid expressing firefly luciferase under the control of the complete IFN beta promoter. Where appropriate, cells were co-transfected with plasmids expressing VF1 or empty vector (pcDNA3.1) along with plasmids expressing cellular proteins (e.g. RIG-I etc. [85]). Empty plasmid was added to ensure each transfection received the same amount of total DNA. To normalize for transfection efficiency and to ensure lack of protein toxicity, the pRLTK Renilla luciferase reporter plasmid was added to each transfection. Samples were lysed 24 hours post transfection in passive lysis buffer (Promega) and activity measured using a dual luciferase reporter assay system (Promega) as described [85].

### *In vivo* virulence analysis

In order to analyze the virulence of wild type and VF1 mutant M1 viruses in mice, age (six to eight weeks, up to16 animals per group) and sex matched animals were orally inoculated (oral gavage) with the WT or the VF1 knockout viruses (diluted in DMEM to a total volume of 100 µl). Control mice (a group of 6) were inoculated with non-infected cell lysates prepared in an identical manner to the virus stocks. Mice were sourced from Taconic (STAT1 -/-) or Harlan (C57BL/6) and verified as MNV free at the beginning of the study. Control mice were verified as MNV free at the end of the study confirming barrier controls were effective. At various times post infection post infection tissue samples were taken post mortem. In addition a fecal sample was taken post mortem. All samples were snap-frozen and either stored in Trizol solution (Invitrogen) or RNA later (Ambion) before the RNA was extracted, as per the manufacturer's instructions. Remaining mice were left for the duration of the experiment or euthanized based on the following humane end points: a 20% loss of bodyweight, the development of severe symptoms (ataxia, moribundity), or the presence of moderate MNV-1 specific symptoms (continued weight loss, discharge from the eye) for 3 consecutive days. These limits were set in order to prevent undue suffering to the animal. In accordance with funding regulations and to minimize the number of animals used in these studies, the data for the control and WT-v inoculated groups of animals in Figure 7 has been reported in a previous study [14]. Note that both the previous experiment and that illustrated in Figure 8 were performed side by side.

### q-RT-PCR quantification of viral load

Tissue samples were stored in Trizol reagent (Invitrogen) or RNa Later (Ambion) and after homogenization RNA was extracted according to the manufacturer instructions. Upon quantification, an aliquot of total RNA from each tissue sample was used for reverse transcription using AMV RT enzyme (Promega) and a primer specific for the genomic RNA of MNV-1 (IC464, CAAACATCTTTCCCTTGTTC). qPCR reactions were prepared using the MESA Blue qPCR MasterMix Plus for SYBR Assay (Eurogentech). Briefly, cDNA was mixed with 2X buffer and primers IC464 and IC465 (TGGACAACGTGGTGAAGGAT) prior to activation by incubation at 95°C for 10 min. Reactions were then subjected to 40 cycles of 94°C, 15 sec; 58°C, 30 sec; 72°C, 30 sec. Viral genome copy number was calculated by interpolation from a standard curve generated using serial dilutions of standard RNA representing nucleotides 1085 to 1986 generated by *in vitro* transcription and extrapolated back to per µg of input RNA. The limit of detection was determined either by the lowest dilution of control standard RNA reproducibly detected in the assay (Figure 7) or by incubating serial dilutions of standard RNA in RNA extracted from the tissues of mock infected animals (Figure 9). An equivalent protocol was used to determine the genome copy number in RNA samples extracted from infected RAW264.7 cells, using 500 ng of input RNA was used.

### Analysis of CXCL10, ISG54 and IFN-Beta levels in infected RAW264.7 cells

Analysis of mRNA levels in RAW264.7 cells was performed at low MOI (0.1 TCID_50_ units/cell). Cells were seeded at 2×10^5^ cells per well in a 24 well dish, grown overnight at 37°C before being infected. RNA was harvested from infected cells at 16, 20 and 24 hours post infection using the GenElute RNA extraction kit (Sigma) as detailed by the manufacturer. RNA was quantified and diluted to a standard concentration before being reverse transcribed using MuMLV RT enzyme (Promega) using an oligo dT primer. SYBR green based qPCR was performed using an ABI 7900 HT real time PCR machine. The MESA Blue (Eurogentec) SYBR master mix was combined with sample cDNAs and optimised high efficiency mouse specific primers for the following genes: HPRT, CXCL10, ISG54 and Interferon Beta (primer details available upon request). Relative mRNA fold change was calculated using the ΔΔCt method e.g. normalising target mRNA levels based on an endogenous control (HPRT) before comparison with mock infected cells at equivalent time points. Where appropriate this fold change was then normalised to the level of MNV RNA detected in each sample in order to provide statistical information on mRNA or protein fold induction relative to MNV RNA and allow for the variance in the susceptibility of RAW264.7 cell clones to MNV infection (data not shown). Data handling and fold change was calculated using the SDS 2.3 and RQ programs from ABI. For the UV inactivation experiments high titre stocks of MNV were cross-linked for 15 minutes under high intensity UV light using a Spectrolinker XL-1500 (Spectronics Corporation). The artificial viral dsRNA analogue poly IC was used to confirm the suitability of RAW264.7 as a model for innate immunity, specifically their capacity to sense dsRNA. polyIC was added directly to the media at a final concentration of 25 µg/ml. RNA was harvested at 24 hours post addition and analysed by qPCR as detailed above. Analysis of IFN-Beta protein levels was performed using a murine IFN-B specific ELISA (PBL Interferon Source) as per manufacturer's instructions. Supernatants from infected or treated cells were centrifuged for 5 minutes at 2,000 rcf before analysis to remove any cellular debris.

### Analysis of the role of ORF4 in control of MNV induced apoptosis

For apoptosis assays, RAW264.7 cells were seeded at 5×10^5^ cells per well of a 24-well plate and grown overnight at 37°C. Cells were infected with an MOI of 5 TCID_50_ per cell or treated with 5 µM staurosporine (Sigma) and at given time points (9, 12, 15, 18 and 21 h post infection) cells were PBS washed and lysed in 1 ml of 1 x cell culture lysis reagent (Promega). 100 µl of lysate was then incubated with 100 µl of Glo3/7 assay reagent (Promega) and after incubating at room temperature for 40 minutes, luminescence was read using a TD20/20 luminometer (Turner Designs). Samples were subsequently analyzed for protein content and the luminescence signal normalised to account for variations in the efficiency of cell lysis. Where UV-inactivated virus was used, virus stocks were UV-inactivated on ice for 20 minutes using a UV-crosslinker at 254 nm (Stratagene). Mock treated stocks were used as controls and were simply kept on ice for the same period of time prior to dilution and use in virus infections.

Levels of cleaved caspase 3, were compared by western blot analysis using antibodies from Cell Signalling Technology. Rabbit polyclonal antibodies generated against the viral polymerase (NS7) and major capsid protein (VP1) were used to control for equal amounts of virus, whereas a mouse monoclonal antibody to GAPDH (Ambion) was used to ensure equal protein loading.

## Supporting Information

Figure S1 **The response of RAW264.7 cells to polyIC and UV inactivated viruses.** IFN-Beta, CXCL10 and ISG54 mRNA and IFN-Beta protein analysis of RAW264.7 cells (A) infected with an equivalent MOI of 0.1 of UV inactivated M1 and WT viruses at 24hpi or (B) treated with 25 µg/ml polyIC for 24 hours. mRNA levels were quantified by qPCR using an endogenous control gene (Hypoxanthine-guanine phosphoribosyltransferase, HPRT). Expression of the respective mRNAs was then calculated using the ΔΔCt method to compare infected and mock infected cells. IFN-Beta protein secretion was quantified by murine IFN-B specific ELISA in the supernatants of treated/infected cells. Relative fold change was calculated using mock infected samples taken at comparable time points.
**(TIF)**

Figure S2 **VF1 expression contributes to viral virulence.** Age and sex matched STAT1-/- mice were inoculated by oral gavage with 10,000 TCID_50_ of low passage, sequence verified, wild-type (WT-v) or VF1 knockout (M1-v) viruses generated using a virulent backbone cDNA construct. As a measure of the severity of clinical disease, body weight was measured on a daily basis and expressed as percentage of the weight on day 0 prior to inoculation. Mock infected animals were orally inoculated with a control lysate preparation, generated as described in the materials and methods. The error bars represent the mean and standard error for each group. Statistical analysis was performed using two-way ANOVA and Bonferroni post tests (WT-v versus M1-v).
**(TIF)**

Figure S3 **Reversion of VF1 expression does not readily occur during replication in vivo.** Sequence analysis of viral RNA extracted from tissues isolated from M1-v infected moribund animals on day 5 (A) and day 7 (B). The identity and weight loss of the animals is also illustrated in Figure 7. RNA was extracted from the relevant tissues and subjected to RT-PCR amplification of the region encoding the mutated sequence of VF1. MLN and SI refer to the mesenteric lymph node and small intestine respectively. (C) The sensitivity of consensus sequencing of the VF1 region was confirmed by combining cDNA constructs of with the WT or M1 viruses at various combinations prior to PCR and sequencing. The positions of the mutation site is boxed. Consensus sequencing could readily detect reversion in 25% of the population.
**(TIF)**

Figure S4 **CXCL10 and ISG54 responses in MNV infected STAT-/- mice at 3 days post infection.** Tissues were harvested from STAT1-/- mice infected with 1000 TCID-50 of MNV-1 (CW1.P1) at 3 days post infection. Relative fold induction of the CXCL10 and ISG54 mRNAs was calculated for each tissue separately, through comparison with mRNA levels in tissues from a mock/uninfected mouse (represented by the green line). This analysis was performed using the ΔΔCt qPCR method with the cellular gene HPRT used as an endogenous control. The data shown is from two separate animals, with the bar referring to the mean fold induction detected in each instance.
**(TIF)**
