# Supplementary figures and images for: Norovirus Regulation of the Innate Immune Response and Apoptosis Occurs via the Product of the Alternative Open Reading Frame 4
Source: PLoS Pathog. 2011 Dec 8;7(12):e1002413. doi: 10.1371/journal.ppat.1002413 (PMC3234229; doi:10.1371/journal.ppat.1002413)

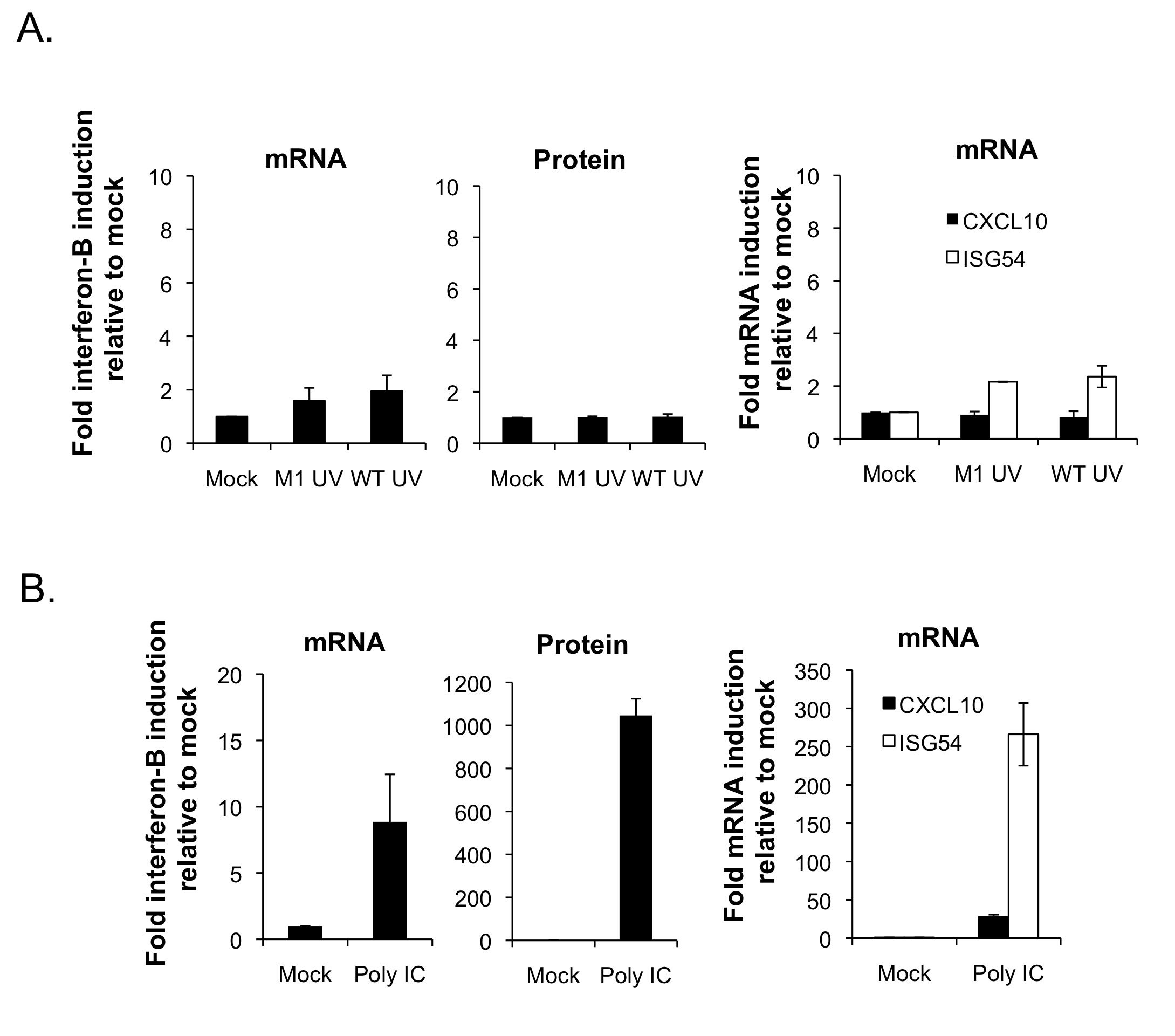

Supplement: Figure S1 — The response of RAW264.7 cells to polyIC and UV inactivated viruses. IFN-Beta, CXCL10 and ISG54 mRNA and IFN-Beta protein analysis of RAW264.7 cells (A) infected with an equivalent MOI of 0.1 of UV inactivated M1 and WT viruses at 24hpi or (B) treated with 25 µg/ml polyIC for 24 hours. mRNA levels were quantified by qPCR using an endogenous control gene (Hypoxanthine-guanine phosphoribosyltransferase, HPRT). Expression of the respective mRNAs was then calculated using the ΔΔCt method to compare infected and mock infected cells. IFN-Beta protein secretion was quantified by murine IFN-B specific ELISA in the supernatants of treated/infected cells. Relative fold change was calculated using mock infected samples taken at comparable time points. (TIF) [file ppat.1002413.s001.tif]

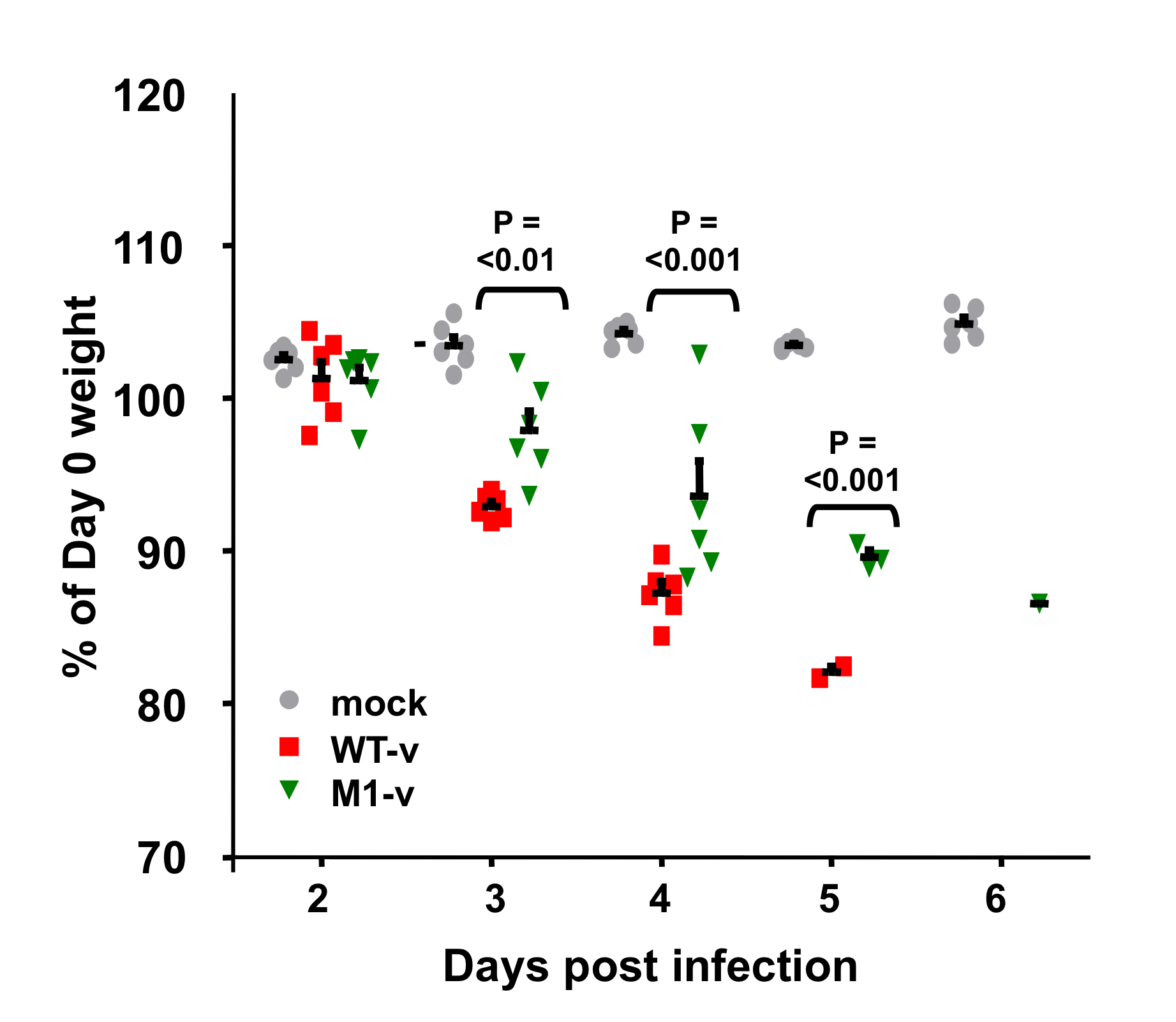

Supplement: Figure S2 — VF1 expression contributes to viral virulence. Age and sex matched STAT1-/- mice were inoculated by oral gavage with 10,000 TCID50 of low passage, sequence verified, wild-type (WT-v) or VF1 knockout (M1-v) viruses generated using a virulent backbone cDNA construct. As a measure of the severity of clinical disease, body weight was measured on a daily basis and expressed as percentage of the weight on day 0 prior to inoculation. Mock infected animals were orally inoculated with a control lysate preparation, generated as described in the materials and methods. The error bars represent the mean and standard error for each group. Statistical analysis was performed using two-way ANOVA and Bonferroni post tests (WT-v versus M1-v). (TIF) [file ppat.1002413.s002.tif]

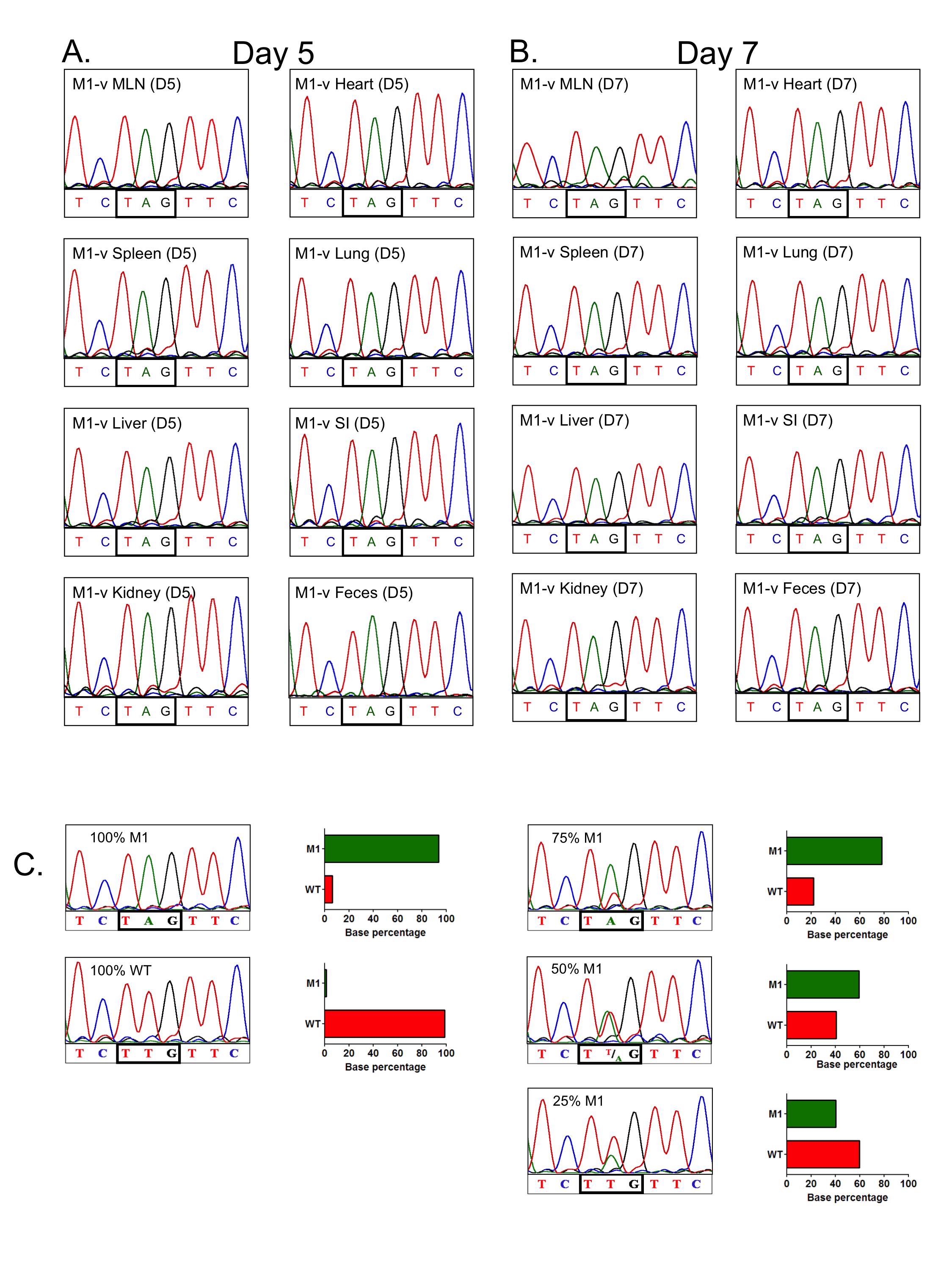

Supplement: Figure S3 — Reversion of VF1 expression does not readily occur during replication in vivo. Sequence analysis of viral RNA extracted from tissues isolated from M1-v infected moribund animals on day 5 (A) and day 7 (B). The identity and weight loss of the animals is also illustrated in Figure 7. RNA was extracted from the relevant tissues and subjected to RT-PCR amplification of the region encoding the mutated sequence of VF1. MLN and SI refer to the mesenteric lymph node and small intestine respectively. (C) The sensitivity of consensus sequencing of the VF1 region was confirmed by combining cDNA constructs of with the WT or M1 viruses at various combinations prior to PCR and sequencing. The positions of the mutation site is boxed. Consensus sequencing could readily detect reversion in 25% of the population. (TIF) [file ppat.1002413.s003.tif]

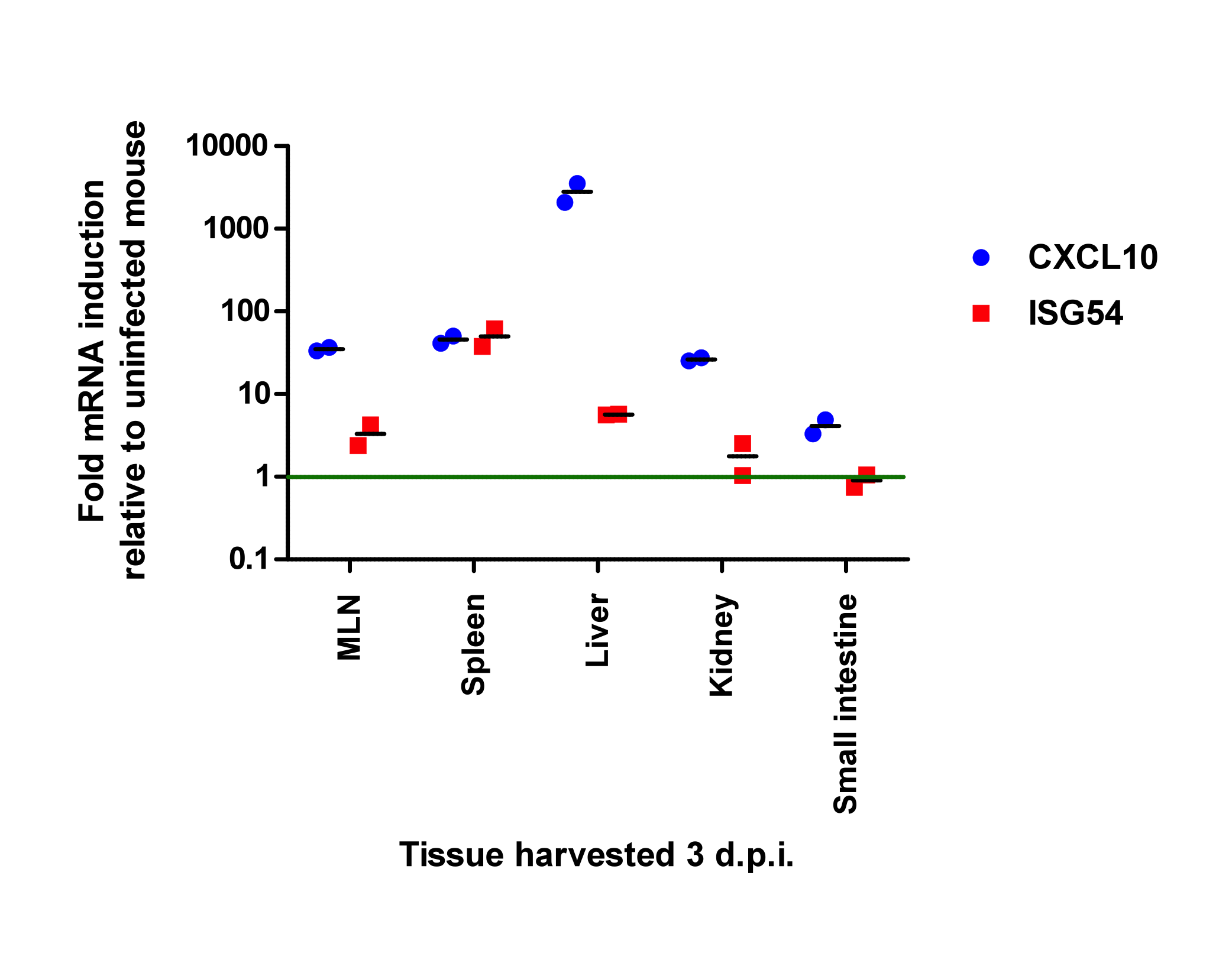

Supplement: Figure S4 — CXCL10 and ISG54 responses in MNV infected STAT-/- mice at 3 days post infection. Tissues were harvested from STAT1-/- mice infected with 1000 TCID-50 of MNV-1 (CW1.P1) at 3 days post infection. Relative fold induction of the CXCL10 and ISG54 mRNAs was calculated for each tissue separately, through comparison with mRNA levels in tissues from a mock/uninfected mouse (represented by the green line). This analysis was performed using the ΔΔCt qPCR method with the cellular gene HPRT used as an endogenous control. The data shown is from two separate animals, with the bar referring to the mean fold induction detected in each instance. (TIF) [file ppat.1002413.s004.tif]
